# Sorting Nexin 27 Regulates the Lysosomal Degradation of Aquaporin-2 Protein in the Kidney Collecting Duct

**DOI:** 10.3390/cells9051208

**Published:** 2020-05-13

**Authors:** Hyo-Jung Choi, Hyo-Ju Jang, Euijung Park, Stine Julie Tingskov, Rikke Nørregaard, Hyun Jun Jung, Tae-Hwan Kwon

**Affiliations:** 1 Department of Biochemistry and Cell Biology, School of Medicine, Kyungpook National University, Taegu 41944, Korea; sky1339@nate.com (H.-J.C.); gywn0001@naver.com (H.-J.J.); euijung.park.84@gmail.com (E.P.); 2 New Drug Development Center, Daegu-Gyeongbuk Medical Innovation Foundation, Taegu 41061, Korea; 3 BK21 Plus KNU Biomedical Convergence Program, Department of Biomedical Science, School of Medicine, Kyungpook National University, Taegu 41944, Korea; 4 Department of Clinical Medicine, Aarhus University, Aarhus 8200, Denmark; sjt@clin.au.dk (S.J.T.); rn@clin.au.dk (R.N.); 5 Division of Nephrology, Department of Medicine, Johns Hopkins University School of Medicine, Baltimore, MD 21205, USA; hjung24@jhmi.edu

**Keywords:** aquaporin-2, lysosomal degradation, retromer complex, sorting nexin 27

## Abstract

Sorting nexin 27 (SNX27), a PDZ (Postsynaptic density-95/Discs large/Zonula occludens 1) domain-containing protein, cooperates with a retromer complex, which regulates intracellular trafficking and the abundance of membrane proteins. Since the carboxyl terminus of aquaporin-2 (AQP2c) has a class I PDZ-interacting motif (X-T/S-X-Φ), the role of SNX27 in the regulation of AQP2 was studied. Co-immunoprecipitation assay of the rat kidney demonstrated an interaction of SNX27 with AQP2. Glutathione S-transferase (GST) pull-down assays revealed an interaction of the PDZ domain of SNX27 with AQP2c. Immunocytochemistry of HeLa cells co-transfected with FLAG-SNX27 and hemagglutinin (HA)-AQP2 also revealed co-localization throughout the cytoplasm. When the PDZ domain was deleted, punctate HA-AQP2 labeling was localized in the perinuclear region. The labeling was intensively overlaid by Lysotracker staining but not by GM130 labeling, a cis-Golgi marker. In rat kidneys and primary cultured inner medullary collecting duct cells, the subcellular redistribution of SNX27 was similar to AQP2 under 1-deamino-8-D-arginine vasopressin (dDAVP) stimulation/withdrawal. Cell surface biotinylation assay showed that dDAVP-induced AQP2 translocation to the apical plasma membrane was unaffected after SNX27 knockdown in mpkCCD cells. In contrast, the dDAVP-induced AQP2 protein abundance was significantly attenuated without changes in AQP2 mRNA expression. Moreover, the AQP2 protein abundance was markedly declined during the dDAVP withdrawal period after stimulation under SNX27 knockdown, which was inhibited by lysosome inhibitors. Autophagy was induced after SNX27 knockdown in mpkCCD cells. Lithium-induced nephrogenic diabetes insipidus in rats revealed a significant downregulation of SNX27 in the kidney inner medulla. Taken together, the PDZ domain-containing SNX27 interacts with AQP2 and depletion of SNX27 contributes to the autophagy-lysosomal degradation of AQP2.

## 1. Introduction

Aquaporin-2 (AQP2) is a water channel protein expressed in the kidney connecting tubule and collecting ducts and plays an essential role in vasopressin-induced water reabsorption and urinary concentration [1,2,3,4]. A number of studies demonstrated that dysregulation of AQP2 plays a critical role in water balance disorders, e.g., nephrogenic diabetes insipidus (NDI) and systemic water retention [5,6]. AQP2 is regulated by vasopressin on a short-term or a long-term basis for collecting duct water reabsorption. Short-term regulation is dependent on the translocation of AQP2 from subapical vesicles to the apical plasma membrane, associated with phosphorylation of the serine residues (serine 256, serine 264, and serine 269) in the carboxyl terminus of AQP2 (AQP2c) [1,7,8,9]. Long-term regulation is based on the prolonged half-life of AQP2 protein and the induction of *Aqp2* gene transcription [2,6,10,11].

The AQP2c is subjected to post-translational modification, e.g., phosphorylation and ubiquitination [6,12,13,14]. In particular, the last four-amino acid sequence in the AQP2c (residues 268–271) corresponds to a class I PDZ (Postsynaptic density-95/Discs large/Zonula occludens 1) domain-binding motif [X-(S/T)-X-Φ, where X is any amino acid and Φ is any hydrophobic residue] [15,16,17,18]. A previous study revealed that signal-induced proliferation-associated gene-1 (SPA-1) is a PDZ domain-containing protein that mediates AQP2 trafficking to the apical plasma membrane [15]. Depletion of SPA-1 reduced apical AQP2 expression, indicating that SPA-1 is likely to be directly bound to AQP2 and regulates AQP2 trafficking [15]. Moreover, signal-induced proliferation-associated 1 like 1 (Sipa1I1), another PDZ domain-containing protein, mediates AQP2 endocytosis in the absence of vasopressin [19].

The retromer complex is a crucial component of the endosomal protein sorting machinery [20,21,22]. The complex is composed of the cargo-selective trimer Vps26-Vps29-Vps35 (hVps26, hVps29, and hVps35 in human) and the membrane-associated heterodimer of two sorting nexin (SNX) proteins Vps5-Vps17 (SNX1 and SNX2 in human) [20]. In mammals, the retromer complex is recruited to endosomes, where it facilitates cargo retrieval from endosomes to the trans Golgi network. Moreover, the retromer complex contributes to the cargo sorting in the early endosomes before cargo delivery to several intracellular compartments, including the recycling of membrane proteins to the plasma membrane. We previously demonstrated that vacuolar protein sorting-associated protein 35 (Vps35) interacts with the AQP2c, and the depletion of Vps35 was associated with decreased AQP2 trafficking and increased lysosomal degradation of AQP2 [23]. Consistently, a recent study also demonstrated that AQP2 accumulated in the recycling endosomes without apical AQP2 trafficking in response to Vps35 knockdown [24].

The sorting nexins belong to a family of proteins characterized by the presence of a PX (Phox homology) domain. They are expressed throughout the endosomal system, participating in several trafficking pathways [25]. Among the sorting nexins, sorting nexin 27 (SNX27) is the only member having a PDZ domain and is one of three sorting nexins containing an atypical FERM (C-terminal 4.1/ezrin/radixin/moesin)-like domain [26]. Previous studies have shown that SNX27 cooperates with the retromer complex by interacting directly with the retromer subunit Vps26 of the Vps26:Vps29:Vps35 trimer and plays a role in the regulation of endosomal recycling and protein abundance [27,28,29]. SNX27 was known to interact with transmembrane proteins containing Asn-Pro-Xaa-Tyr (NPxY) sequences and also with the transmembrane proteins having the class I PDZ domain-binding motifs [X-(S/T)-X-Φ] through its PDZ domain [30]. After interacting with target transmembrane proteins having the PDZ domain-binding motif, SNX27 cooperates with the retromer complex, preventing the entry of transmembrane proteins into the lysosomal pathway, and activating the retromer-tubule-based recycling to the plasma membrane [31]. Since AQP2c has a class I PDZ domain-binding motif, we hypothesized that SNX27 interacts with AQP2c through its PDZ domain, and regulates intracellular trafficking as well as the protein abundance of AQP2. The aim of the present study was, therefore, to examine the role of SNX27 in the vasopressin-mediated regulation of AQP2 in the kidney collecting duct cells, which provides new insights into the AQP2 regulatory mechanism.

## 2. Materials and Methods

### 2.1. cDNA Construction of Rat SNX27

**Figure 1:**
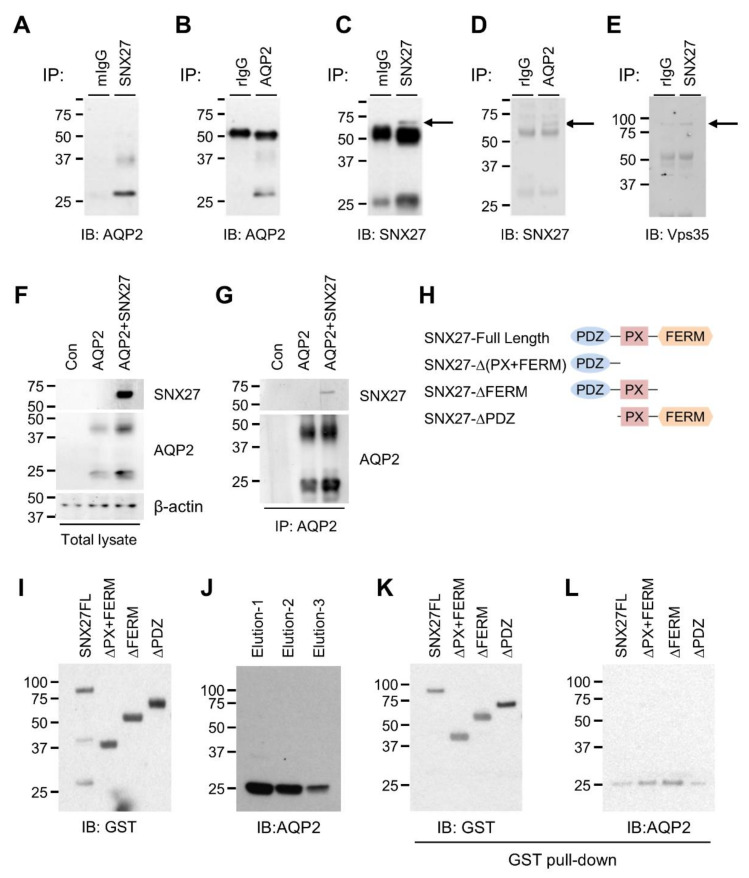
Co-immunoprecipitation of aquaporin-2 (AQP2) and sorting nexin 27 (SNX27). (**A**,**B**) Immunoblotting of AQP2 in pull-down samples from rat kidney inner medulla tubule suspension using pre-immune immunoglobulin G (IgG) of mouse (mIgG), Dynabead M-280 with anti-SNX27 antibody, pre-immune IgG of rabbit (rIgG), or Dynabead M-280 with anti-AQP2 antibody, respectively. (**C**,**D**) Immunoblotting of SNX27 in pull-down samples from rat kidney inner medulla tubule suspension using pre-immune IgG of mouse (mIgG), Dynabead M-280 with anti-SNX27 antibody, pre-immune IgG of rabbit (rIgG), or Dynabead M-280 with anti-AQP2 antibody, respectively. (**E**) Immunoblotting of vacuolar protein sorting-associated protein 35 (Vps35) in pull-down samples from rat kidney inner medulla tubule suspension using pre-immune IgG of rabbit (rIgG) or Dynabead M-280 with anti-SNX27 antibody, respectively. (**F**) Human Embryonic Kidney 293T (HEK293T) cells were transiently expressed with hemagglutinin (HA)-tagged AQP2 (full length) plasmid alone or both HA-tagged AQP2 (full length) and FLAG-tagged SNX27 (full length) plasmid. Immunoblotting of AQP2 and SNX27. In the control condition (Con), cells were transfected only with p3XFLAG-CMV-10 and pcDNA3.1-HA. (**G**) Cell lysates were immunoprecipitated with anti-HA antibody and immunoblotted with anti-AQP2 antibody and anti-SNX27 antibody. (**H**) Schematic representation of SNX27 constructs. (**I**) Immunoblotting using anti-glutathione S-transferase (GST) antibody after purification of GST-tagged SNX27 constructs. (**J**) Immunoblotting using anti-AQP2 antibody after purification of histidine (His)-tagged carboxyl terminus of AQP2 (AQP2c). (**K**,**L**) GST-SNX27 fusion proteins were incubated with His-tagged AQP2c proteins and precipitated using Glutathione Sepharose 4B beads. Precipitates were immunoblotted with anti-glutathione S-transferase (GST) or anti-AQP2 antibody.

The SNX27 gene was amplified by PCR using primers from the cDNA (complementary DNA) of rat kidney inner medulla (Table 1). The amplified PCR products were cloned into the pGEX-4T-1 and p3XFLAG-CMV-10 vectors. cDNA constructs of SNX27 were generated according to the endonuclease recognition sites (Figure 1E) [32]: SNX27-full length (1–539 amino acids), SNX27 lacking PX and FERM domains [(SNX27-Δ(PX+FERM), 1–156 amino acid residue], SNX27 lacking an FERM domain [(SNX27-ΔFERM), 1–266 amino acid residue], and SNX27 lacking a PDZ domain [(SNX27-ΔPDZ), 158–539 amino acid residue]. These constructs were cloned into the *BamHI* and *XhoI* site of the pGEX-4T-1 vector and *HindIII* of the p3XFLAG-CMV-10 vector. The final constructs were confirmed by Sanger sequencing (Macrogen, Korea).

### 2.2. Purification of Recombinant SNX27 Protein

*Escherichia coli* strain BL21 (DE3) was transformed for protein expression of pGEX-4T-1-rat SNX27-full length, pGEX-4T-1-rat SNX27-Δ(PX+FERM), pGEX-4T-1-rat SNX27-ΔFERM, and pGEX-4T-1-rat SNX27-ΔPDZ, respectively. Transformed *E. coli* BL21 cells were inoculated in 200 mL of Luria-Bertani (LB) broth with 100 mg/mL ampicillin at 37 °C for 3 h and protein expression was induced by the addition of isopropyl β-d-1-thiogalactopyranoside (IPTG, 1 mM) at 18 °C overnight. After induction, *E. coli* BL21 cells were harvested by centrifugation (4000× *g*, 20 min, 4 °C). The pellet was resuspended in GST (glutathione S-transferase) binding buffer (140 mM NaCl, 2.7 mM KCl, 10 mM Na_2_HPO_4_, and 1.8 mM KH_2_PO_4_, pH 7.3) containing 0.5% Triton X-100 and protease inhibitor (100 mM PMSF, 0.4 μg/mL Leupeptin, and 0.1 mg/mL Pefabloc) and disrupted by sonication on ice. The supernatant separated by centrifugation (13,200 rpm, 30 min, 4 °C) was applied to 500 μL of Glutathione Sepharose 4B beads (GE Healthcare) at room temperature for 30 min. After washing six times with 2.5 mL of GST binding buffer, proteins bound to the resin were eluted three times with 500 μL of GST elution buffer (50 mM Tris-HCl, 10 mM reduced glutathione, pH 8.0). Following elution, proteins were examined by immunoblotting performed on Bolt^TM^ 4–12% Bis-Tris Plus Gels (Thermo Fisher Scientific, Waltham, MA). The calculated mass of each GST-bound protein of pGEX-4T-1-rat SNX27-full length was ~85 kDa, pGEX-4T-1-rat SNX27-Δ(PX+FERM) was ~43 kDa, pGEX-4T-1-rat SNX27-ΔFERM was ~55 kDa, and pGEX-4T-1-rat SNX27-ΔPDZ was ~68 kDa, all of which were confirmed by immunoblotting using GST antibody (91G1, 1:1000, Cell Signaling, Danvers, MA, USA, Figure 1F).

### 2.3. Purification of Recombinant His_6X_-FLAG-AQP2c Fusion Protein

Recombinant AQP2c was made in *Escherichia coli* BL21 (DE3) by exploiting a pET32 TrxA fusion system, as we previously demonstrated [23]. *E. coli* BL21 cells transformed with pET32aM-His_6X_-FLAG-AQP2c vector [23] were inoculated in 200 mL of LB broth with 100 mg/mL ampicillin. After incubation at 37 °C for 3 h, the protein expression was induced by isopropyl β-d-1-thiogalactopyranoside (IPTG, 1 mM) at 18 °C overnight. The cells were harvested, and the pellet was sonicated [23]. The soluble fraction of the lysates was separated by centrifugation (12,000 rpm, 10 min, 4 °C) and applied to Ni-NTA agarose (QIAGEN, Hilden, Germany) at 4 °C for 30 min. The agaroses were washed by washing buffer (20 mM Tris∙HCl, 500 mM NaCl, 10 mM imidazole, pH 8.0) and the bound proteins were eluted three times by 500 μL of elution buffer (20 mM Tris∙HCl, 500 mM NaCl, 250 mM imidazole, pH 8.0) after incubation at room temperature for 10 min.

### 2.4. Interaction of Recombinant SNX27 Proteins and AQP2c Protein In Vitro

The purified recombinant SNX27 proteins were poured into Poly-Prep Chromatography Columns (BioRad, Hercules, CA, USA) filled out with 500 μL of Glutathione Sepharose 4B beads. Interactions between recombinant SNX27 proteins and beads were carried out at 4 °C for 30 min. The beads were washed five times with 2 mL of GST binding buffer and the purified recombinant His_6X_-FLAG-AQP2c proteins were applied to columns at 4 °C for 20 h. The bound proteins were kept at room temperature for 15 min and then washed four times with the same buffer. The proteins bound to the beads were eluted three times after incubation at room temperature for 10 min with 500 μL of GST elution buffer each time.

### 2.5. Co-Immunoprecipitation

The animal protocols were approved by the Animal Care and Use Committee of Kyungpook National University (KNU 2012-10). For the immunoprecipitation experiments, inner medullary collecting duct (IMCD) tubules were prepared from rat kidneys [23,33,34]. Briefly, the inner medulla (IM) was isolated from male Sprague-Dawley rats (200–250 g, Charles River, Seongnam, Korea), and was dissected, minced, and digested in IMCD suspension buffer (250 mM sucrose and 10 mM triethanolamine, pH 7.4) containing collagenase B (3 mg/mL) and hyaluronidase (2 mg/mL). The IMCD tubule suspension was then continuously agitated in a water bath at 37 °C for 90 min. Thereafter, the IMCD tubule suspension was centrifuged at 60× *g* for 30 s to separate the IMCD-enriched fraction (pellet) and the non-IMCD fraction (supernatant). The IMCD-enriched fraction was collected and lysed using ice-cold immunoprecipitation (IP) lysis buffer (87787, Thermo Fisher Scientific) with protease inhibitors and was incubated on ice for 5 min. The lysates were centrifuged at 13,000× *g* for 10 min to remove the cell debris at 4 °C and supernatant was used for the co-immunoprecipitation analysis. Magnetic beads (Dynal M-280, Thermo Fisher Scientific) were resuspended, transferred to tubes, and washed three times with phosphate-buffered saline (PBS). Anti-rabbit normal immunoglobulin G (IgG) (sc-2027, Santa Cruz Biotechnology, Dallas, TX), anti-mouse normal IgG (sc-2025, Santa Cruz Biotechnology), anti-AQP2 (host: rabbit, AB3274, Merck Millipore, Burlington, MA), and anti-SNX27 (host: mouse, ab77799, abcam, Cambridge, UK) antibodies were added to the washed beads, respectively. The mixture of antibody-beads and IMCD lysate was shaken and incubated with a slow rotator overnight at 4 °C. After washing, 1X sample buffer containing dithiothreitol (DTT) was added and incubated for 30 min at 65 °C. Samples were collected by a magnetic pull-down. For an additional immunoprecipitation assay of SNX27 and Vps35 in the rat kidney IMCD tubule suspension, anti-SNX27 (host: rabbit, NBP1-45283, Novus Biologicals, Centennial, CO) and anti-Vps35 antibody (host: mouse, sc-374372, Santa Cruz Biotechnology) were used.

HEK293T cells were cultured in high glucose Dulbecco’s modified eagle’s medium (DMEM-high glucose, HyClone, Logan, UT) with 0.1% penicillin-streptomycin and 10% heat-inactivated fetal bovine serum (FBS). Cells were seeded on a 100-mm culture dish, and when they reached 80–90% confluency, cells were transfected with p3XFLAG-CMV-10 (5 μg) and pcDNA3.1-HA (5 μg) for the control condition or p3XFLAG-CMV-10-SNX27 (5 μg) and pcDNA3.1-HA-AQP2 (5 μg) using Lipofectamine 2000 reagent in DMEM-high glucose media with 10% FBS. After 4 h of transfection, the medium was changed to DMEM-high glucose with 0.1% penicillin-streptomycin and 10% FBS and cultured for 24 h. Cells were harvested by centrifugation at 1500 rpm for 3 min. Cell pellets were lysed using ice-cold IP lysis buffer (87787, Thermo Fisher Scientific) with protease inhibitors (1.15 mM PMSF, 0.4 μg/mL Leupeptin, 0.1 mg/mL Pefabloc) and incubated on ice for 5 min. The lysates were centrifuged at 13,000× *g* for 10 min to remove the cell debris at 4 °C and the supernatant was applied to the co-immunoprecipitation analysis. Anti-AQP2 (host: rabbit, AB3274, Merck Millipore, Burlington, MA) and anti-SNX27 antibody (host: mouse, ab77799, abcam) were added to the washed magnetic beads (Dynal M-280, Thermo Fisher Scientific), respectively. The mixture of antibody-HEK293T cell supernatant-bead was rotated slowly by the rotator overnight at 4 °C. After washing, 1X sample buffer containing DTT was added and incubated for 30 min at 65 °C. Samples were collected by magnetic pull-down.

### 2.6. Immunofluorescence Microscopy and Quantification of Co-Localization

Immunofluorescence analysis was performed on cultured cells and kidney tissue sections from paraffin-embedded preparations (2 um thickness), as previously described [33,35]. For double-immunolabeling of AQP2 and SNX27 on kidney sections, deparaffinized sections were incubated with rabbit anti-AQP2 polyclonal antibody (1:200, AB3274, Merck Millipore), and mouse anti-SNX27 monoclonal antibody (1:200, ab77799, abcam) followed by Alexa Fluor 488-conjugated goat anti-rabbit IgG or Alexa Fluor 594-conjugated goat anti-mouse IgG. Immunofluorescence microscopy was performed using a laser scanning confocal microscope (Zeiss LSM 800; Jena, Germany).

To examine the interaction between PDZ domain-containing SNX27 and PDZ domain-binding motif-containing AQP2, we performed double-immunolabeling of the FLAG-tagged SNX27 and HA-tagged AQP2 in HeLa cells. HeLa cells were cultured in DMEM supplied with 10% FBS at 37 °C in a 5% CO_2_ incubator and transfected with Lipofectamine 2000 reagent for FLAG-tagged SNX27 truncated constructs and HA-tagged AQP2. For double-immunolabeling of HA-AQP2 and FLAG-SNX27, cells were incubated with mouse anti-HA antibody (1:200, 2367S, Cell signaling), and rabbit-anti FLAG antibody (1:200, 14793, Cell signaling), followed by Alexa Fluor 488-conjugated goat anti-rabbit IgG or Alexa Fluor 647-conjugated goat anti-mouse IgG. For the staining of intracellular lysosomes, HeLa cells were stained with Lysotracker Red DND-99 (1 μM, L7528, Molecular Probes) for 1 h before immunolabeling of HA-tagged AQP2. For double labeling of AQP2 and the Golgi structure, mouse anti-HA antibody (1:200, 2367S, Cell signaling) and rabbit-anti GM130 antibody (1:200, ab52649, abcam), a cis-Golgi marker [36], were used. As previously demonstrated [37], a quantitative analysis of co-localization was performed on the confocal sections showing maximum signals of SNX27, AQP2, Lysotracker, GM130, GFP, and RFP using Image J (NIH). JACoP (Just another co-localization plugin) was used to calculate the Pearson’s coefficient (https://imagej.nih.gov/ij/plugins/track/jacop2.html).

### 2.7. Primary Culture of Inner Medullary Collecting Duct (IMCD) Cells from the Rat Kidney

The animal protocols were approved by the Animal Care and Use Committee of Kyungpook National University (KNU 2012-10). Primary cultures enriched in IMCD cells were prepared from pathogen-free male Sprague-Dawley rats (200–250 g, Charles River, Seongnam, Korea), as we previously demonstrated [23,33].

### 2.8. Real-Time Quantitative PCR

mpkCCDc14 cells were transfected with control-siRNA or SNX27-siRNA (50 nM) using a reverse transfection method and grown on semi-permeable filters of 6-well transwell plates for 9 days [38]. Total RNA purification was performed by Direct-zol^TM^ RNA MiniPrep (Zymo Research, Irvine, CA), according to the manufacturer’s instruction, and cDNAs were synthesized using 1 ug of total RNA by the Prime Script cDNA Synthesis kit (Takara Shuzo Co., Otsu, Shiga, Japan). The relative expression of the AQP2 and SNX27 mRNA was determined by real-time quantitative PCR (RT-qPCR), using a QuantiTect SYBR Green PCR Kit (QIAGEN), respectively. The PCR reaction was performed at 95 °C for 10 min followed by 30 cycles at 95 °C for 15 s, 58 °C for 30 s, and 72 °C for 30 s. β-actin was used as an internal control, and the threshold was set by 0.02 to determine the threshold cycle (Ct) value. The fold enrichment was calculated as 2^−[ΔCt(AQP2 or SNX27)−ΔCt(β-actin)]^. RT-qPCR of mRNA was carried out using Rotor-Gene-A (QIAGEN), and each sample was tested in duplicate. Primers of mRNA were designed by Origene and purchased from Cosmogenetech (Seoul, Korea).

### 2.9. Semiquantitative Immunoblotting in mpkCCDc14 Cells with SNX27 Knockdown

To examine the effects of SNX27 knockdown on the 1-deamino-8-d-arginine vasopressin (dDAVP)-induced changes in AQP2 protein abundance, mpkCCDc14 cells transfected with SNX27-siRNA (50 nM) using Dharmafect (Dharmacon, Lafayette, CO, USA) were seeded on semipermeable filters of the Transwell system (0.4-µm pore size, Transwell^®^ Permeable Supports, catalog no. 3450, Corning, CORNING, NY, USA) for 7 days until polarization and then incubated in serum-free and hormone-deprived medium for another 24 h before dDAVP treatment. Then, dDAVP (10^−9^ M, Sigma, St. Louis, MO, USA) was applied to the basolateral side of mpkCCDc14 cells for the last 24 h. To examine the effects of chloroquine, bafilomycin, or MG-132 treatment on AQP2 protein abundance during the withdrawal period of dDAVP stimulation under the SNX27 knockdown, mpkCCDc14 cells were treated with vehicle or chloroquine (10^−4^ M, Sigma, a blocker of the lysosomal pathway of protein degradation), bafilomycin (10^−7^ M, BD, Franklin Lakes, NJ, USA; a blocker of the lysosomal pathway), or MG-132 (10^−4^ M, Calbiochem, San Diego, CA, USA; an inhibitor of proteasomal degradation) in the hormone-deprived medium for 3 h during the withdrawal period of dDAVP stimulation. Changes in the protein abundance of AQP2, SNX27, and LC3 (#2775, Cell Signaling) were analyzed by semiquantitative immunoblotting, as we previously demonstrated [23,33,39].

### 2.10. Cell Surface Biotinylation Assay

Cell surface biotinylation was done in mpkCCDc14 cells, as we described previously [33]. mpkCCDc14 cells were washed with ice-cold PBS-CM (10 mM PBS containing 1 mM CaCl_2_ and 0.1 mM MgCl_2_, pH 7.5) and treated for 45 min at 4 °C in ice-cold biotinylation buffer (10 mM triethanolamine, 2 mM CaCl_2_, 125 mM NaCl, pH 8.9) containing 1 mg/mL sulfosuccinimidyl 2-(biotinamido)-ethyl-1,3-dithiopropionate (Sulfo-NHS-SS-biotin, Thermo Scientific, Rockford, IL, USA) on the apical side. Cells were then washed with quenching buffer (50 mM Tris-HCl in PBS-CM, pH 8.0), followed by washes with PBS-CM. Lysis buffer (150 mM NaCl, 5 mM ethylenediaminetetraacetic acid (EDTA), 50 mM Tris-HCl, 1% Triton X-100, 1.15 mM PMSF, 0.4 μg/mL Leupeptin, 0.1 mg/mL Pefabloc, 0.1 μM okadaic acid, 1 mM Na_3_VO_4_, and 25 mM NaF) was added and lysates were sonicated for 7 s at 20% of the amplitude twice. Lysates were centrifuged at 10,000× *g* for 5 min at 4 °C. The supernatant was transferred to columns where Neutravidin agarose resin (200 μL) had been loaded (Thermo Scientific) and incubated overnight at 4 °C. After being washed with PBS containing protease inhibitors, 1X sample buffer containing DTT was added to the column and samples were incubated for 60 min at room temperature. Samples were heated at 65 °C for 10 min.

### 2.11. Lithium-Induced Nephrogenic Diabetes Insipidus in Rats

The animal protocols were performed in accordance with the Danish National Guidelines for the care and handling of animals. The protocols were approved by the Institute of Clinical Medicine, Aarhus University, according to the licenses for the use of experimental animals issued by the Danish Ministry of Justice (Approval no. 2015-15-0201-00658). The study was performed on male Sprague-Dawley rats, weighing 175–215 g. Rats were housed with a 12:12-h light-dark cycle, a temperature of 21 ± 2 °C, and a humidity of 55 ± 2%. The rats had free access to standard rat chow (Altromin, Lage, Germany) and water during the experiment. The rats used as controls were fed an unsupplemented standard rat chow for 14 days (*n* = 8). Lithium-treated rats were fed a standard diet where lithium chloride (LiCl; Sigma-Aldrich, Copenhagen, Denmark) was added in a concentration of 40 mmol/kg dry food for 14 days (*n* = 10). This concentration of LiCl in the food results in a level of serum lithium at therapeutic levels [40]. After 14 days, the rats were sacrificed, and the kidneys were removed and prepared for semiquantitative immunoblotting and immunohistochemistry [40]. These male rats were used in our previous study, and the functional data after the lithium treatment were published [40]. For immunolabeling, the removed kidneys were fixed in 4% paraformaldehyde, embedded in paraffin, and processed for immunoperoxidase labeling, as previously described [41]. Sections were incubated with mouse anti-SNX27 monoclonal antibody (ab77799, abcam) at 4 °C overnight, then incubated for one hour with horseradish peroxidase-conjugated secondary antibody (polyclonal goat anti-mouse IgG (P447, Dako, Glostrup, Denmark). Conventional light microscopy was performed using an Olympus BX50 microscope and CellSens Imaging software (Olympus).

### 2.12. Statistical Analysis

Values are presented as means ± SE. Comparisons were made by the unpaired *t*-test (between two groups) or by one-way ANOVA followed by Bonferroni’s multiple-comparison test (more than two groups). Multiple comparisons tests were only applied when a significant difference was determined by ANOVA. *P* values of <0.05 were considered significant.

## 3. Results

### 3.1. Interaction between AQP2 and SNX27

To examine the endogenous interaction between AQP2 and SNX27, we performed immunoprecipitation assay from a rat kidney IMCD tubule suspension. Immunoblot analysis demonstrated that AQP2 was detected in pull-down samples using an anti-SNX27 mouse monoclonal antibody (Figure 1A) or an anti-AQP2 rabbit polyclonal antibody (Figure 1B). In the control experiments, AQP2 was not detected in the pull-down samples using pre-immune IgG of a normal mouse or rabbit (Figure 1A,B). Immunoblot analysis with an anti-SNX27 antibody also detected SNX27 in the pull-down samples using an anti-SNX27 mouse monoclonal antibody (indicated by an arrow in Figure 1C) or anti-AQP2 rabbit polyclonal antibody (indicated by an arrow in Figure 1D), indicating that AQP2 and SNX27 formed a complex in the kidney IMCD. In the control experiments, SNX27 was not detected in the pull-down samples using pre-immune IgG of a normal mouse or rabbit (Figure 1C,D). Additionally, immunoblot analysis with an anti-Vps35 antibody detected Vps35 in the pull-down samples using an anti-SNX27 rabbit antibody (indicated by an arrow in Figure 1E).

Interaction between AQP2 and SNX27 was also demonstrated in HEK293T cells. A plasmid of the AQP2 cDNA (full length) that was aligned in-frame with an HA sequence and a plasmid of the SNX27 cDNA (full length) aligned in-frame with a 3XFlag sequence were constructed. Then, HEK293T cells were transiently expressed with either HA-tagged AQP2-full length or HA-tagged AQP2-full length and FLAG-tagged SNX27-full length. Immunoblot analysis of the total cell lysates demonstrated that AQP2 or both AQP2 and SNX27 were identified in the cells expressing either HA-tagged AQP2-full length or HA-tagged AQP2-full length and FLAG-tagged SNX27-full length, respectively (Figure 1F). In the pull-down samples using an anti-HA mouse monoclonal antibody, both AQP2 and SNX27 were identified in the cells expressing both HA-tagged AQP2-full length and FLAG-tagged SNX27-full length (Figure 1G), further indicating a complex formation between AQP2 and SNX27.

To determine which domain of SNX27 interacts with the carboxyl terminus of AQP2 (AQP2c) directly, we conducted an in vitro pull-down assay. GST-SNX27 constructs (SNX27-Full Length, SNX27-Δ(PX+FERM), SNX27-ΔFERM, and SNX27-ΔPDZ) were generated (Figure 1H). The purified recombinant GST-fusion SNX27 proteins were subjected to the interaction with the purified recombinant His_6X_-Flag-AQP2c protein in Poly-Prep Chromatography Columns (BioRad) and eluted proteins were confirmed by immunoblotting of GST and AQP2 (Figure 1I,J). In Figure 1K,L, the GST pull-down assays revealed that the recombinant SNX27 proteins having the PDZ domain (SNX27-Full Length, SNX27-Δ(PX+FERM), SNX27-ΔFERM) were well detected by the AQP2 antibody (Figure 1L). In contrast, SNX27 protein lacking the PDZ domain (SNX27-ΔPDZ) was not readily detected by the AQP2 antibody (Figure 1L), although the loading concentration of protein (i.e., the density of GST immunoblot) was higher than others in the GST pull-down (Figure 1K). The findings indicated that the PDZ domain of SNX27 directly interacted with AQP2c, which has the PDZ domain-binding motif.

### 3.2. Immunocytochemistry and Immunohistochemistry of AQP2 and SNX27 in HeLa Cells and Rat Kidneys

**Figure 2:**
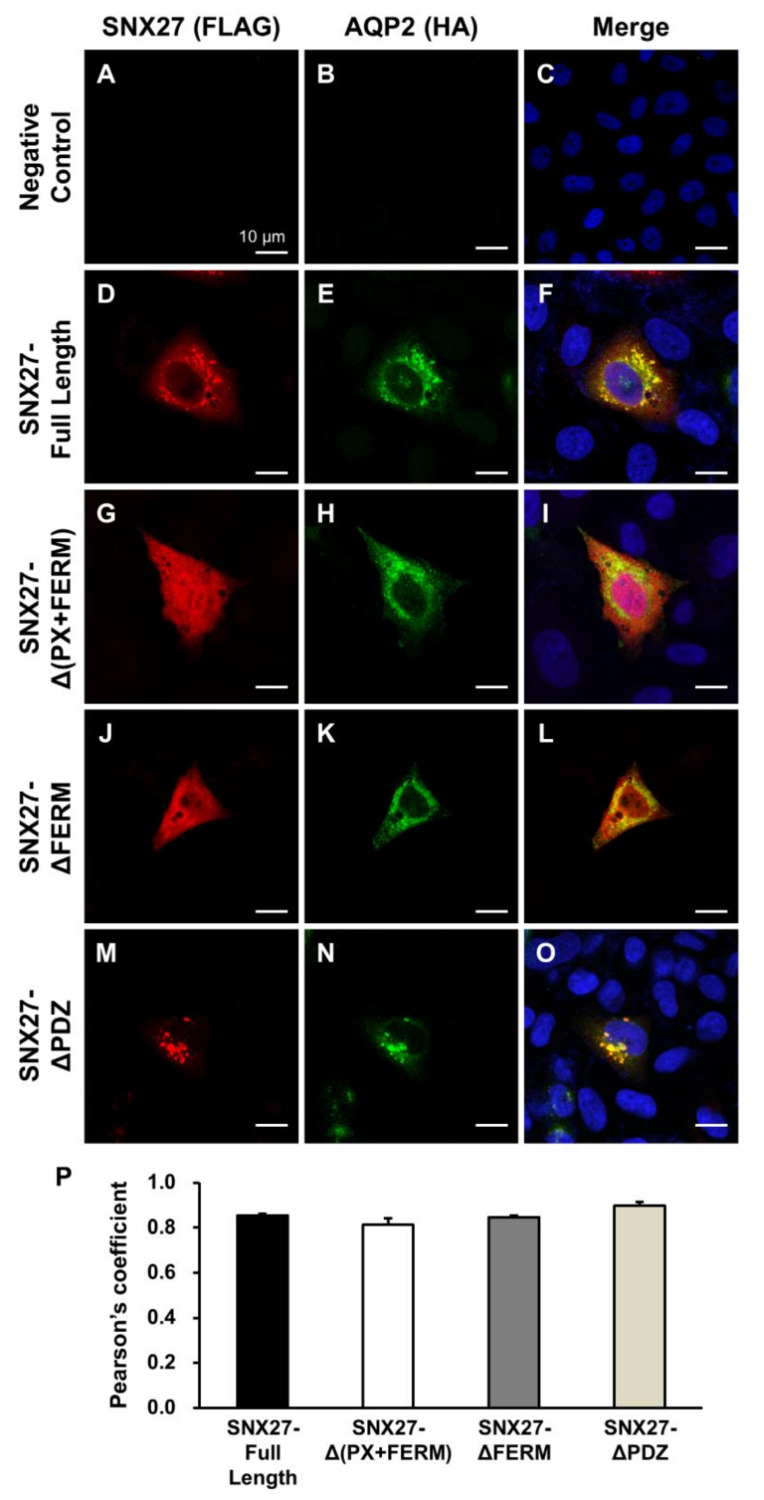
Immunofluorescence of SNX27 and AQP2 in HeLa cells. FLAG-tagged SNX27 and HA-tagged AQP2 constructs were transiently transfected into HeLa cells and immunolabeling of FLAG and HA was done. A negative control study revealed no immunolabeling of FLAG and HA in the HeLa cells transiently transfected with SNX27-full length and HA-tagged AQP2 constructs, which were incubated only with secondary antibody (omitting the incubation of primary antibodies) (**A**–**C**). SNX27 (SNX27-Full Length, Δ(PX+FERM), and ΔFERM) and AQP2 were co-localized throughout the cytoplasm in HeLa cells (**D**–**L**). In contrast, when the PDZ domain was deleted in SNX27 (SNX27-ΔPDZ), SNX27 and AQP2 accumulated in a punctate pattern, and more eccentrically localized in the perinuclear region of the HeLa cells (**M**–**O**). (**P**) Co-localization of SNX and AQP2 was analyzed by calculation of the Pearson’s coefficient. Graphs express means ± SE (>20 cells per group; three independent experiments). Scale bars, 10 μm.

Based on the interaction between PDZ domain-containing SNX27 and PDZ domain-binding motif-containing AQP2, we examined the intracellular localization of FLAG-tagged SNX27 and HA-tagged AQP2 in HeLa cells (Figure 2). Both FLAG-tagged SNX27 constructs (SNX27-Full Length, SNX27-Δ(PX+FERM), SNX27-ΔFERM, and SNX27-ΔPDZ, respectively) and HA-tagged AQP2 constructs were transiently transfected into HeLa cells and immunocytochemistry of FLAG and HA was done. Immunolabeling using antibodies against tagged proteins (FLAG and HA) was performed to exclude the localization of endogenous expression of SNX27 in HeLa cells. In the control experiments, laser scanning immunofluorescence confocal microscopy revealed no immunolabeling of FLAG and HA in the HeLa cells transiently transfected with SNX27-full length and HA-tagged AQP2 constructs (negative control in Figure 2A–C). Immunocytochemistry demonstrated that AQP2 co-localized with SNX27-full length (Figure 2D–F). Specifically, immunolabeling of FLAG-tagged SNX27 (SNX-Full Length, SNX27-Δ(PX+FERM), and SNX27-ΔFERM) was dispersed throughout the cytoplasm in HeLa cells, where HA-tagged AQP2 was co-localized (Figure 2D–L). In contrast, when the PDZ domain was deleted (SNX27-ΔPDZ), FLAG-tagged SNX27 and HA-tagged AQP2 revealed a different pattern: Both were observed in a punctate pattern, and more eccentrically localized in the perinuclear region of the HeLa cells (Figure 2M–O). Co-localization of SNX and AQP2 was analyzed by calculation of the Pearson’s coefficient. The Pearson’s coefficient of FLAG and HA signals was similar in all four groups (Figure 2P).

**Figure 3:**
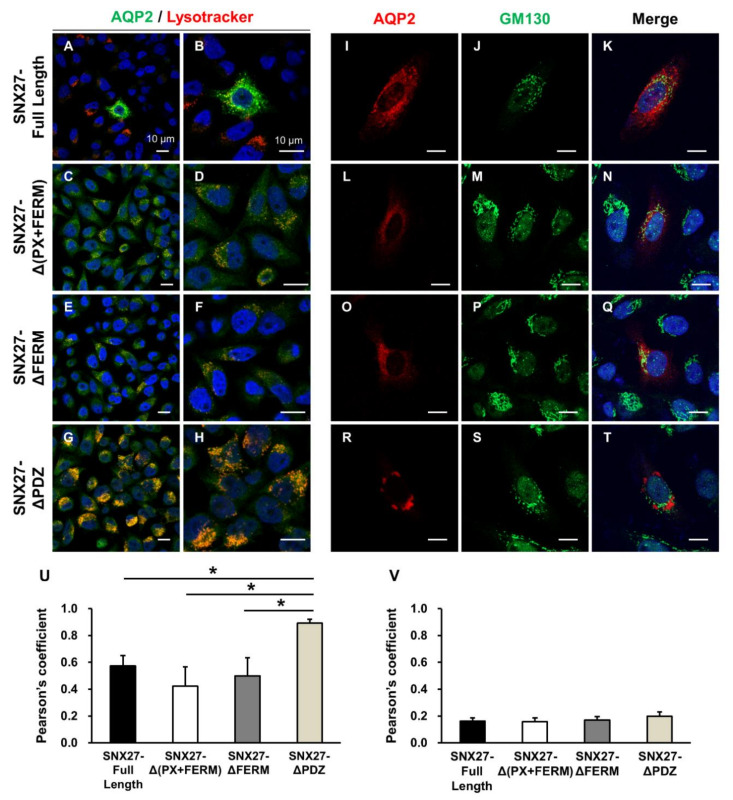
Immunofluorescence of AQP2, Lysotracker, or GM130 in HeLa cells. FLAG-tagged SNX27 and HA-tagged AQP2 constructs were transiently transfected into HeLa cells. (**A**–**H**) Cells were stained with Lysotracker (red), followed by immunolabeling of HA (green). Diffuse cytoplasmic HA labeling (i.e., AQP2 in panels (**A**–**F**)) was not or weakly overlaid by Lysotracker Red staining in the cytoplasm of HeLa cells with PDZ domain-expressing SNX27 (**A**–**F**). In contrast, when the PDZ domain was deleted in SNX27 (SNX27-ΔPDZ), the punctate AQP2 labeling was observed, which was intensively overlaid by Lysotracker Red staining (**G**,**H**). Panels (**B**,**D**,**F**,**H**) are zoomed-in images of panels (**A**,**C**,**E**,**G**), respectively. (**I**–**T**) Cells were co-immunolabeled with anti-HA and anti-GM130 antibodies. HA-AQP2 labeling was not overlaid by GM130 labeling in the presence or absence of the PDZ domain in SNX27. (**U**,**V**) Co-localization of HA-tagged AQP2 and Lysotracker (**U**) or HA-tagged AQP2 and GM130 (**V**) was analyzed by calculation of the Pearson’s coefficient. Graphs express means ± SE (>30 cells per group; three independent experiments). * *p* < 0.05. Scale bars, 10 μm.

To further determine the subcellular localization of the observed punctate HA-tagged AQP2 labeling in HeLa cells transfected with both FLAG-tagged SNX27 (SNX27-ΔPDZ) and HA-tagged AQP2 constructs, cells were stained with Lysotracker, a lysosome marker, or were immunolabeled with anti-GM130 antibody, a cis-Golgi marker. FLAG-tagged SNX27 constructs (SNX27-Full Length, SNX27-Δ(PX+FERM), SNX27-ΔFERM, and SNX27-ΔPDZ, respectively) and HA-tagged AQP2 constructs were transiently transfected into HeLa cells. HeLa cells were stained with Lysotracker for 1 h before the immunolabeling of HA. The results showed that HA labeling (i.e., AQP2) was not or weakly overlaid by Lysotracker staining in the cytoplasm of HeLa cells with PDZ domain-expressing SNX27 (SNX27-Full Length, Δ(PX+FERM), and ΔFERM) and AQP2 (Figure 3A–F). In contrast, when the PDZ domain was deleted in SNX27 (SNX27-ΔPDZ), punctate AQP2 labeling was observed, which was intensively overlaid by Lysotracker staining (Figure 3G,H). This finding was further supported by the significant increase in the co-localization coefficient of HA and Lysotracker (Figure 3U). This finding suggested that the observed punctate co-labeling of FLAG-tagged SNX27 and HA-tagged AQP2 when the PDZ domain was deleted in SNX27 (Figure 2M–O and Figure 3G,H) was associated with a lysosomal compartment in the cells. On the contrary, HA-AQP2 labeling was not overlaid by GM130 labeling in the presence or absence of the PDZ domain in SNX27 (Figure 3I–T). This finding was consistent with the low co-localization coefficient of HA and GM130 in all four groups (Figure 3V).

**Figure 4:**
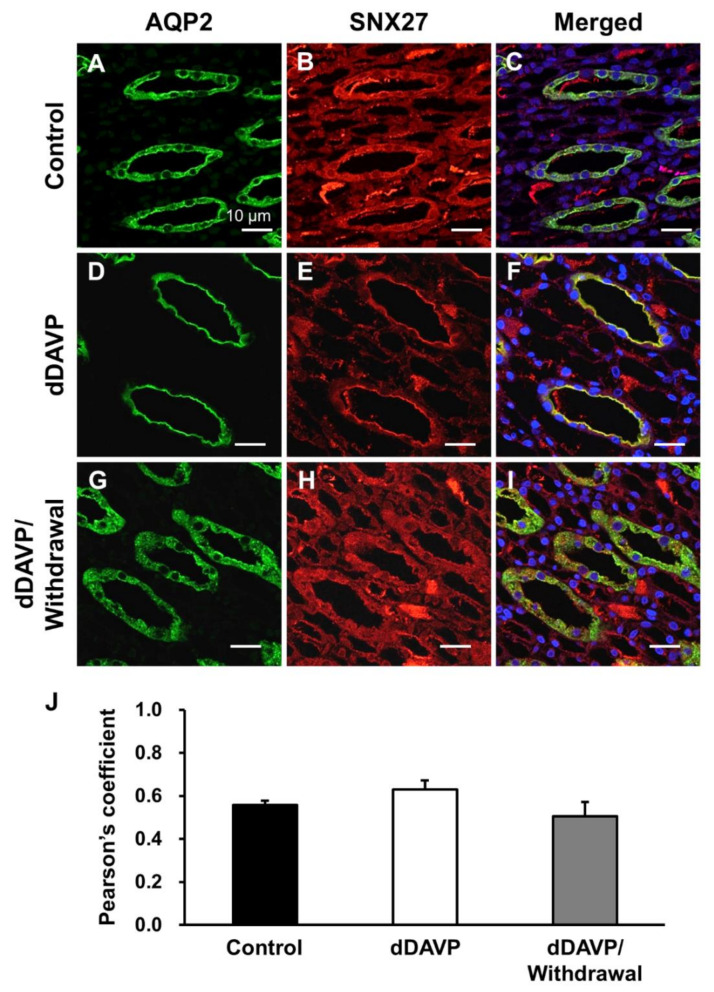
Immunofluorescence microscopy of AQP2 and SNX27 in the rat kidney inner medulla. Immunofluorescence labeling of AQP2 in the inner medullary collecting duct cells of the kidney from vehicle-treated control rats (Control, (**A**)), rats with dDAVP infusion for 5 days (dDAVP, (**D**)), and rats with dDAVP withdrawal for 3 h after dDAVP infusion for 5 days (dDAVP/withdrawal, (**G**)). SNX27 immunolabeling in the kidneys from vehicle-treated rats (**B**), rats with dDAVP infusion for 5 days (D5d, (**E**)), and rats with dDAVP withdrawal for 3 h after dDAVP infusion for 5 days (D5d-3 h, (**H**)). Immunofluorescence labeling of AQP2 and SXN27 was merged (**C**,**F**,**I**). (**J**) Co-localization of AQP2 and SNX27 was analyzed by calculation of the Pearson’s coefficient. Graphs express means ± SE (>200 cells in the collecting ducts per group; two independent experiments). Scale bars, 10 μm.

Next, we performed immunohistochemistry of SNX27 and AQP2 in rat kidneys. In our previous study [14], AQP2 was highly expressed in the apical plasma membrane of the rat kidney after dDAVP infusion for 5 days, whereas AQP2 expression in the apical plasma membrane declined and AQP2 was internalized during the withdrawal period of dDAVP stimulation. In the present study, immunofluorescence microscopy of AQP2 and SNX27 was carried out on the same kidney tissue sections obtained from our previous study [14]. In the kidney inner medullary collecting duct (IMCD) cells of vehicle-treated control rats, AQP2 and SNX27 were mainly localized in the cytoplasm (Figure 4A–C). AQP2 was translocated to the apical plasma membrane after the 5 day-dDAVP infusion in rats (dDAVP, Figure 4D), and SNX27 labeling was also mainly seen in the apical plasma membrane (Figure 4E,F). After the removal of dDAVP stimulation, AQP2 and SNX27 were diffusely labeled again in the cytoplasm (dDAVP/withdrawal, Figure 4G–I). The co-localization coefficient of AQP2 and SNX27 was not significantly changed but persistent before and after dDAVP treatment in the kidney sections (Figure 4J). In the cortical and outer medullary collecting ducts, the intracellular localization of both AQP2 and SNX27 was similar to the findings observed in the IMCD (not shown).

**Figure 5:**
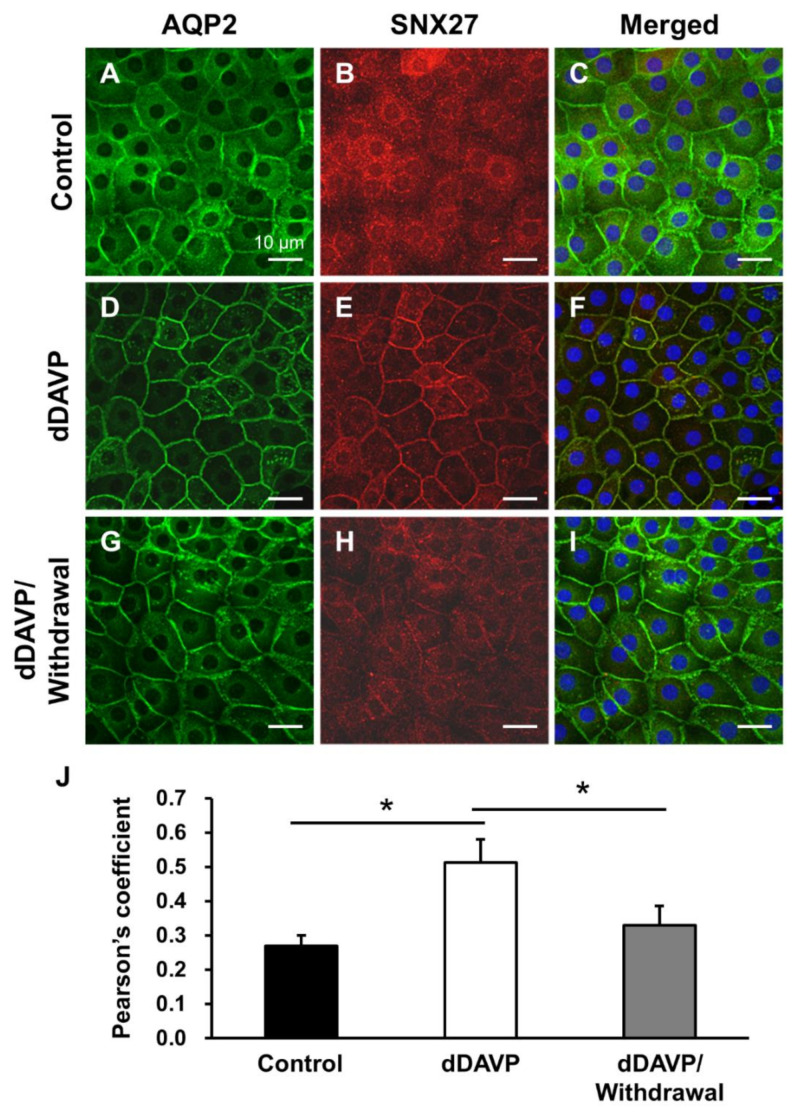
Immunofluorescence microscopy of AQP2 and SNX27 in primary cultured inner medullary collecting duct (IMCD) cells of the rat kidney. The immunofluorescence labeling of AQP2 and SNX27 in vehicle-treated IMCD cells ((**A**,**B**) Control), dDAVP (10^−9^ M)-treated IMCD cells for 24 h ((**D**,**E**) dDAVP), and IMCD cells with dDAVP withdrawal for 3 h after dDAVP (10^−9^ M)-treatment for 24 h ((**G**,**H**) dDAVP-3 h). Immunofluorescence labeling of AQP2 and SXN27 was merged (**C**,**F**,**I**). (**J**) Co-localization of AQP2 and SNX27 was analyzed by calculation of the Pearson’s coefficient. Graphs express means ± SE (>200 cells per group; two independent experiments). * *p* < 0.05. Scale bars, 10 μm.

The experiments of dDAVP stimulation and withdrawal were also done in primary cultured IMCD cells of the rat kidney. Primary cultured IMCD cells were seeded into an 8-well chamber slide and maintained for 3 days. On day 3, IMCD cells were starved in serum-free medium for 24 h, and on day 4, cells were treated with dDAVP (10^−9^ M) for another 24 h at 37 °C. The changes in the immunolocalization of AQP2 and SNX27 (Figure 5A–I) were similar but not identical to the changes observed in the tissue sections of the rat kidney (Figure 4A–I). The cytoplasmic labeling of AQP2 and SNX27 in vehicle-treated IMCD cells (Figure 5A–C) was translocated to the plasma membranes after dDAVP treatment (Figure 5D–F), which was internalized again after the removal of dDAVP stimulation (Figure 5G–I). However, AQP2 immunolabeling was also observed in the plasma membrane of the cells in addition to the cytoplasm in the vehicle-treated control group and dDAVP withdrawal group (Figure 5A,G), whereas SNX27 was mainly localized intracellularly in both conditions (Figure 5B,H). In contrast, both AQP2 and SNX27 were exclusively co-localized in the plasma membrane in response to dDAVP stimulation (Figure 5D,E). Consistent with this, the co-localization coefficient of AQP2 and SNX27 in IMCD cells was significantly higher after dDVAP treatment compared to vehicle-treated IMCD cells or IMCD cells with dDAVP withdrawal for 3 h after dDAVP treatment (Figure 5J). The observed difference in the AQP2 immunolabeling density in the plasma membrane between kidney sections (Figure 4) and primary cultured IMCD cells (Figure 5), particularly in the vehicle-treated control group and the dDAVP withdrawal group, is likely to contribute to the observed difference in the co-localization coefficients.

### 3.3. Changes in AQP2 Protein Abundance in mpkCCDc14 Cells with siRNA-Mediated SNX27 Knockdown

**Figure 6:**
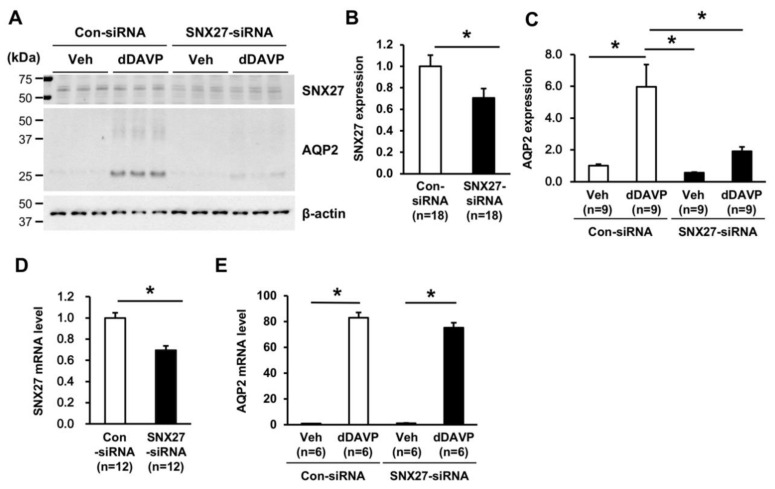
Semiquantitative immunoblotting and quantitative real-time PCR of SNX27 and AQP2 in mpkCCDc14 cells with siRNA-mediated SNX27 knockdown. (**A**–**C**) Semiquantitative immunoblotting of SNX27 (~65 kDa) and AQP2 (~29 kDa and ~35–50 kDa) in total cell lysates from mpkCCDc14 cells treated with vehicle or dDAVP (10^−9^ M) for 24 h under control siRNA or SNX27-siRNA transfection. *n* indicates the number of cell preparations from three independent experiments. (**D**,**E**) SNX27 and AQP2 mRNA level in mpkCCDc14 cells treated with vehicle or dDAVP (10^−9^ M) for 24 h under control siRNA or SNX27-siRNA transfection. *n* indicates the number of cell preparations from two independent experiments. * *p* < 0.05.

Based on the protein–protein interaction and co-localization between SNX27 and AQP2, we examined whether the depletion of SNX27 affects AQP2 expression. SNX27-siRNA treatment in mpkCCDc14 cells significantly decreased SNX27 protein abundance (71 ± 9% of control siRNA-treated group, *p* < 0.05, Figure 6A,B), compared to control siRNA-treated cells. dDAVP treatment (10^−9^ M for 24 h) significantly induced AQP2 protein abundance in mpkCCDc14 cells treated with control siRNA (596 ± 140% of vehicle-treated cells, *p* < 0.05, Figure 6A,C). In contrast, dDAVP-induced AQP2 upregulation was significantly blunted in the cells with siRNA-mediated knockdown of SNX27 (191 ± 28% of vehicle-treated cells under control siRNA, *p* < 0.05 when compared to the control-siRNA group with dDAVP stimulation, Figure 6A,C). Additionally, quantitative real-time PCR (qRT-PCR) was performed to determine whether the decreased dDAVP response in AQP2 protein abundance under the depletion of SNX27 was associated with a change in the AQP2 mRNA level. SNX27-siRNA treatment in mpkCCDc14 cells significantly decreased SNX27 mRNA expression (69 ± 2% of control-siRNA-treated group, *p* < 0.05, Figure 6D). However, SNX27-siRNA treatment did not affect the dDAVP-induced AQP2 mRNA expression in mpkCCDc14 cells (Figure 6E), suggesting that the blunted response in AQP2 protein abundance after dDAVP treatment under SNX27 knockdown was not likely to be mediated by an inhibition of *Aqp2* gene transcription.

**Figure 7:**
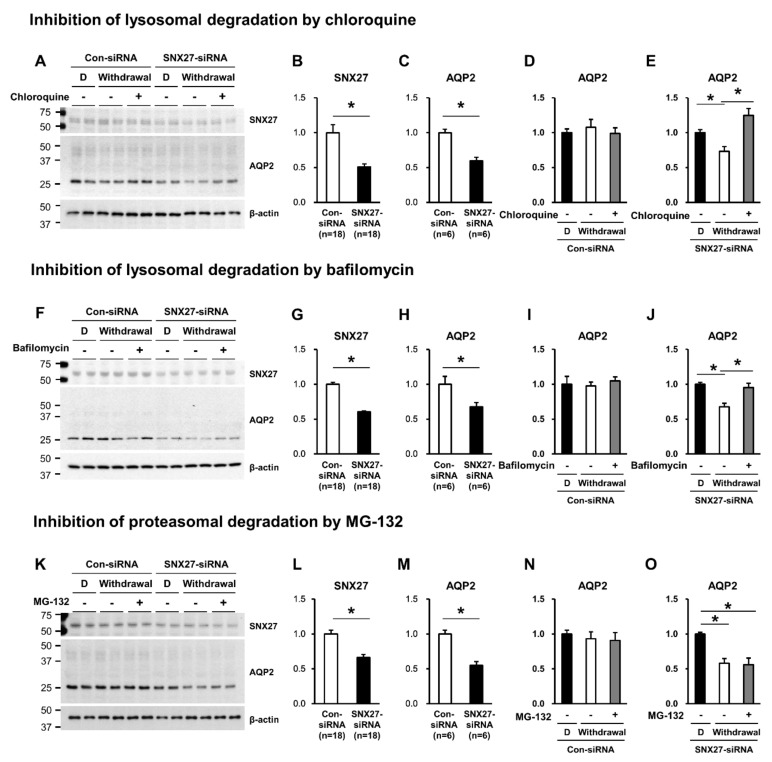
Semiquantitative immunoblotting of SNX27 (**A**,**B**; **F**,**G**; and **K**,**L**) and AQP2 (**A**,**C**; **F**,**H**; and **K**,**M**) in total cell lysates from mpkCCDc14 cells transfected with control-siRNA or SNX27-siRNA. Semiquantitative immunoblotting of AQP2 in total cell lysates from mpkCCDc14 cells transfected with control siRNA or SNX27-siRNA subjected to 24-h dDAVP stimulation (10^−9^ M) or 3-h withdrawal (Withdrawal) after dDAVP stimulation (10^−9^ M, 24 h) in the absence (-) or the presence (+) of chloroquine (10^−4^ M, for the last 3 h, (**A**,**D**,**E**)), bafilomycin (10^−7^ M, for the last 3 h, (**F**,**I**,**J**)), or MG-132 treatment (MG-132, 10^−6^ M, for the last 3 h, (**K**,**N**,**O**)). *n* indicates the number of cell preparations from three independent experiments. D, dDAVP. * *p* < 0.05.

Accordingly, we examined whether the blunted dDAVP response in AQP2 protein abundance under the SNX27 knockdown (Figure 6A,C) was associated with the diminished protein half-life due to enhanced degradation of AQP2 via lysosome and/or proteasome. We previously demonstrated that AQP2 degradation in primary cultured IMCD cells of the rat kidney was significantly attenuated by MG-132 (a proteasome inhibitor) or chloroquine (a blocker of the lysosomal pathway of protein degradation) treatment [14], indicating that AQP2 degradation is mediated by both proteasomal and lysosomal pathways. We examined the effects of chloroquine or bafilomycin (a blocker of the lysosomal degradation) or MG-132 (a proteasome inhibitor) treatment on the changes in AQP2 protein abundance. The changes in AQP2 protein abundance were examined during the dDAVP withdrawal period after stimulation in mpkCCDc14 cells pre-treated with either control siRNA or SNX27-siRNA. SNX27 protein abundance was significantly decreased in SNX27-siRNA-transfected mpkCCDc14 cells, compared to control-siRNA-transfected cells (Figure 7A,B; F,G; and K,L). Consistent with the results in Figure 6, the dDAVP response in AQP2 protein abundance was significantly decreased under the SNX27 knockdown (60 ± 5% of control-siRNA transfected cells, *p* < 0.05, Figure 7A,C; 67 ± 7% of control siRNA transfected cells, *p* < 0.05, Figure 7F,H; and 55 ± 5% of control siRNA transfected cells, *p* < 0.05, Figure 7K,M). mpkCCDc14 cells transfected with control-siRNA demonstrated that AQP2 protein abundance was not changed during 3 h of withdrawal after 24 h of dDAVP stimulation (withdrawal) in the absence or the presence of chloroquine, bafilomycin, or MG-132 cotreatment (Figure 7A,D; F,I; and K,N). In contrast, mpkCCDc14 cells with SNX27 knockdown revealed that AQP2 protein abundance significantly declined during 3 h of withdrawal after 24 h of dDAVP stimulation (73 ± 6% of dDAVP-treated cells under the SNX27 knockdown, *p* < 0.05, Figure 7A,E; 68 ± 5% of dDAVP-treated cells under the SNX27 knockdown, *p* < 0.05, Figure 7F,J; and 58 ± 7% of dDAVP-treated cells under the SNX27 knockdown, *p* < 0.05, Figure 7K,O). Importantly, the decrease in AQP2 protein abundance under the SNX27 knockdown was significantly inhibited by chloroquine or bafilomycin co-treatment (Figure 7A,E; F,J) but not by MG-132 co-treatment (Figure 7K,O). The findings could indicate that SNX27, directly interacting with AQP2 in a PDZ-dependent manner, is likely to regulate the stability of AQP2 protein through regulation of the lysosomal degradation of AQP2.

### 3.4. Changes in dDAVP-Induced Cell Surface Expression of AQP2 in mpkCCDc14 Cells with siRNA-Mediated Knockdown of SNX27

**Figure 8:**
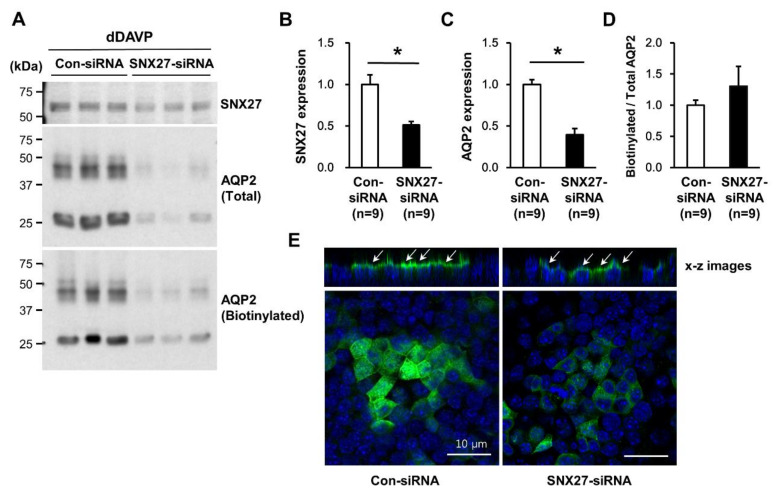
Cell surface biotinylation assay of AQP2 in mpkCCDc14 cells with siRNA-mediated SNX27 knockdown under dDAVP stimulation. (**A**–**C**) Semiquantitative immunoblotting of SNX27 (~65 kDa) and AQP2 (~29 kDa and ~35–50 kDa) in the total cell lysates or biotinylated fraction from mpkCCDc14 cells transfected with control siRNA or SNX27-siRNA. (**A**,**D**) Cell surface biotinylation assay for examining the changes in dDAVP (10^−9^ M, 24 h)-induced AQP2 expression in the apical plasma membrane of the mpkCCDc14 cells. (**E**) Immunofluorescence microscopy of AQP2 in mpkCCDc14 transfected with control siRNA or SNX27-siRNA, followed by dDAVP treatment (10^−9^ M, 24 h). In the x-z images, arrows indicate AQP2 expression in the apical plasma membrane. *n* indicates the number of cell preparations from three independent experiments. * *p* < 0.05 when compared to control siRNA-transfected cells.

To examine whether SNX27 depletion affects dDAVP-induced targeting of AQP2 to the apical plasma membrane, a cell surface biotinylation assay was done in mpkCCDc14 cells with siRNA-mediated knockdown of SNX27. As observed in Figure 6 and Figure 7, the dDAVP-induced increase in AQP2 protein abundance was significantly blunted in mpkCCDc14 cells (40 ± 5% of control siRNA-transfected cells, *p* < 0.05, Figure 8A,C) with siRNA-mediated knockdown of SNX27 (51 ± 1% of control-siRNA-transfected cells, *p* < 0.05, Figure 8A,B). The cell surface biotinylation assay, however, demonstrated that the ratio of AQP2 expression at the biotinylated fraction (apical plasma membrane fraction) to the total fraction was unchanged after SNX27 knockdown (130 ± 30% of control siRNA-transfected cells, not significant, Figure 8A,D). Consistent with this, immunofluorescence microscopy of AQP2 showed that AQP2 immunolabeling in the apical plasma membrane was obvious after dDAVP stimulation (10^−9^ M, 24 h) in mpkCCDc14 cells with SNX27-siRNA-mediated knockdown (x-z images, Figure 8E), despite the markedly decreased labeling intensity of AQP2. This finding suggested that SNX27 depletion was associated with a significant decrease in the vasopressin-induced AQP2 protein abundance, whereas it was not likely to affect the AQP2 trafficking to the apical plasma membrane in the collecting duct cells.

### 3.5. Autophagy in mpkCCDc14 Cells with siRNA-Mediated Knockdown of SNX27 and Changes in SNX27 Protein Abundance in Lithium-Induced Nephrogenic Diabetes Insipidus

**Figure 9:**
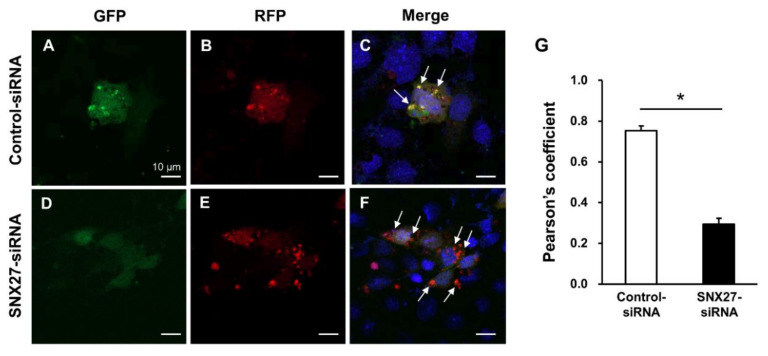

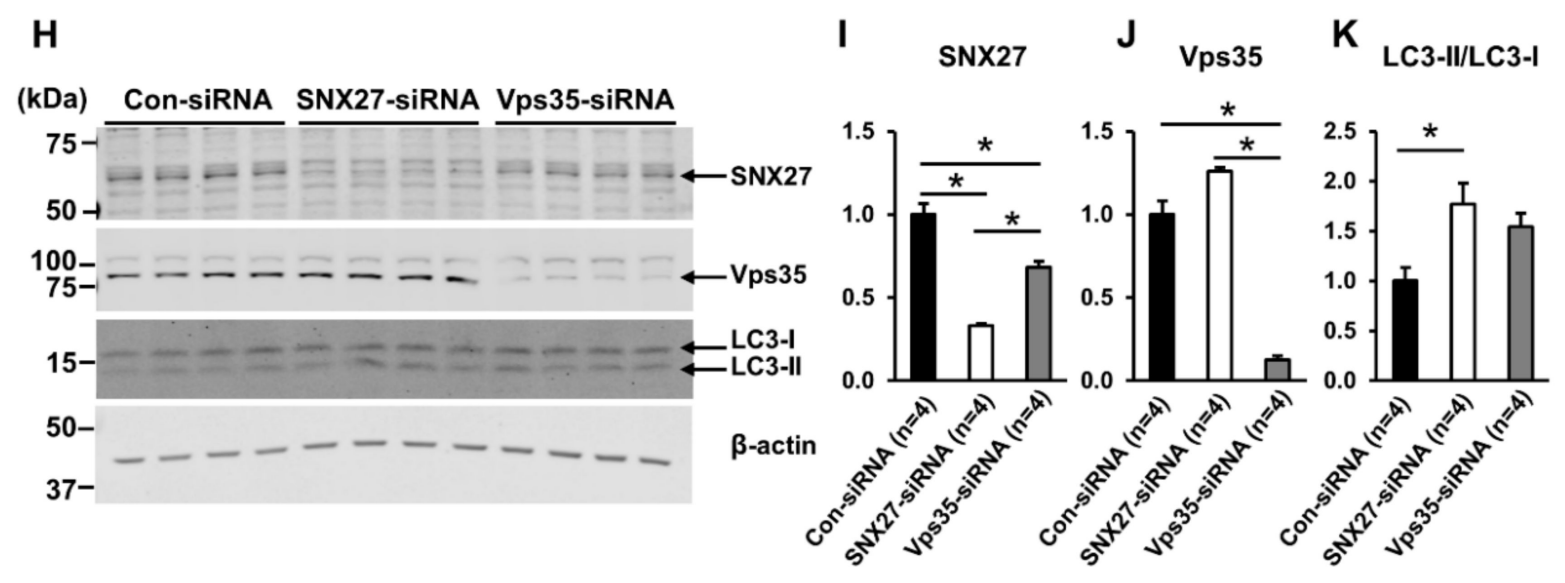
(**A**–**F**) Autophagic flux assessment using mRFP-GFP-LC3 in mpkCCDc14 cells transfected with control siRNA or SNX27-siRNA. To assess autophagic flux from the autophagosomes to lysosomes, RFP-GFP-LC3 plasmid was transfected into mpkCCDc14 cells. (**A**–**C**) GFP and RFP signals co-localize to yellow punctate structures in control siRNA-treated mpkCCDc14 cells (arrows). (**D**–**F**) GFP signal is quenched but RFP signal is seen in SNX27-siRNA-transfected mpkCCDc14 cells (arrows). (**G**) Co-localization of GFP and RFP signals was analyzed by calculation of the Pearson’s coefficient. Graphs express means ± SE (>20 cells per group; two independent experiments). (**H-K**) Semiquantitative immunoblotting of SNX27 (~65 kDa), Vps35 (~90 kDa), and LC3 (LC3-I: ~16 kDa and LC3-II: ~14 kDa) in total cell lysates from mpkCCDc14 cells transfected with control siRNA, SNX27-siRNA, or Vps35-siRNA. *n* indicates the number of cell preparations. * *p* < 0.05. Scale bars, 10 μm.

To evaluate whether SNX27 knockdown is associated with autophagic flux from autophagosome formation, maturation, and fusion with lysosomes, mpkCCDc14 cells were examined using RFP-GFP-LC3 plasmid. mpkCCDc14 cells were transfected with control siRNA or SNX27-siRNA (50 nM) along with the mRFP-GFP-LC3 plasmids using Lipofectamine 2000. Cells were seeded on an 8-well chamber slide (154534, Nunc) and cultured for 72 h. When the mRFP-GFP-LC3-I is converted into mRFP-GFP-LC3-II and inserted into the autophagosomal membrane, RFP and GFP signals co-localize in yellow punctate structures, as demonstrated in the control siRNA-transfected mpkCCDc14 cells (Figure 9A–C, indicated by arrows). In contrast, when autophagosomes fuse with lysosomes, the acidic environment inside the lysosome quenches the GFP fluorescent signal, yet has much less of an effect on RFP [42]. Consistent with this, siRNA-mediated SNX27 knockdown in mpkCCDc14 cells demonstrated a red punctate RFP fluorescence but not much GFP fluorescence (Figure 9D–F, indicated by arrows), indicating that SNX27 knockdown was associated with an induction of the autophagic flux and formation of autolysosomes in mpkCCDc14 cells. The changes in the co-localization of GFP and RFP signals were demonstrated by calculating the Pearson’s coefficient. The data demonstrated that the co-localization coefficient was significantly decreased in SNX27-siRNA-treated cells (Figure 9G).

To examine whether SNX27 or Vps35 knockdown is associated with autophagy, mpkCCDc14 cells transfected with control siRNA (50 nM), SNX27-siRNA (50 nM), or Vps35-siRNA (50 nM) were seeded and cultured on semipermeable filters of the Transwell system (0.4-µm pore size, Transwell^®^ Permeable Supports, catalog no. 3450, Corning) for 4 days. Semiquantitative immunoblotting demonstrated that each siRNA treatment significantly decreased the protein abundance of SNX27 (33 ± 1% of control, *p* < 0.05, Figure 9H,I) or Vps35 (12 ± 1% of control, *p* < 0.05, Figure 9H,J), respectively. Interestingly, the results showed that Vps35 protein abundance was unchanged (126 ± 2% of control, not significant (n.s.), Figure 9H,J) after SNX27 knockdown, whereas SNX27 protein abundance was significantly decreased (68 ± 4% of control, *p* < 0.05, Figure 9H,I) after Vps35 knockdown. Semiquantitative immunoblotting of LC3 demonstrated that the ratio of LC3-II/LC3-I was significantly increased (177 ± 21% of control, *p* < 0.05, Figure 9H,K) after SNX27 knockdown, indicating that the depletion of SNX27 is associated with an increased number of autophagosomes. The depletion of Vps35 tended to demonstrate an increased ratio of LC3-II/LC3-I (154 ± 14% of control, n.s., Figure 9H,K); however, it approximated but did not reach statistical significance.

**Figure 10:**
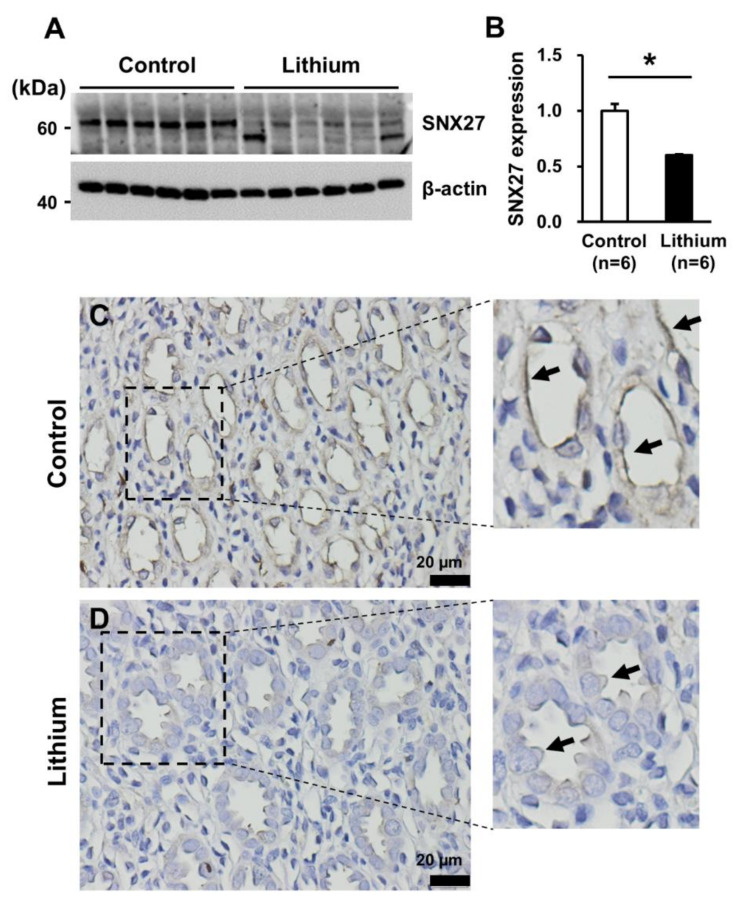
(**A**,**B**) Semiquantitative immunoblotting of SNX27 (~65 kDa) in the kidney inner medulla of lithium-induced nephrogenic diabetes insipidus. (**C**,**D**) Immunoperoxidase labeling of SNX27 (brown color, arrows) in the kidney inner medulla from control rats (control) and rats with lithium-induced nephrogenic diabetes insipidus (Lithium). Magnified images were obtained from the rectangles in panels (**C**,**D**). *n*, number of rats. * *p* < 0.05 when compared to control rats. Scale bars, 20 μm.

Since lithium is known to induce autophagy in a number of cell types [43,44], we directly examined whether lithium-induced NDI is associated with downregulation of SNX27 in the kidney. Semiquantitative immunoblotting of SNX27 was done in the inner medullary protein samples from rats with lithium-induced NDI collected from our previous study [40]. The results demonstrated that SNX27 protein abundance was significantly decreased in the kidney inner medulla of lithium-induced NDI (61 ± 1% of control rats, *p* < 0.05, Figure 10A,B). Immunohistochemistry of rat kidneys also showed weak immunoperoxidase labeling of SNX27 in lithium-induced NDI (arrows in Figure 10D), compared to control rats (arrows in Figure 10C). Immunoperoxidase labeling of SNX27 was seen in both the apical plasma membrane and the cytoplasm of the collecting duct cells in the kidney inner medulla in control rats (Figure 10C). In contrast, the immunoperoxidase labeling intensity of SNX27 in both the apical plasma membrane and the cytoplasm was weaker in lithium-induced NDI (Figure 10D).

## 4. Discussion

We demonstrated that SNX27, a PDZ domain-containing protein, co-localizes with AQP2 in the kidney collecting duct cells in the basal condition as well as in the conditions of dDAVP stimulation and withdrawal. GST pull-down assays in vitro revealed the direct interaction of the PDZ domain of SNX27 with the carboxyl terminus of AQP2 (AQP2c), which has a class I PDZ-interacting motif (X-T/S-X-Φ). The co-immunoprecipitation assay of the rat kidney as well as HEK293T cells transiently expressing AQP2 and SNX27 revealed the complex formation between them, and immunohistochemistry of the rat kidneys also demonstrated co-localization in the kidney collecting ducts.

Interestingly, the data suggested similar binding sites of SNX27 and SPA-1 in AQP2 [15]. SPA-1, a PDZ domain-containing protein, was demonstrated to have GTPase-activating protein activity for AQP2 trafficking [15]. Thus, for the understanding of the function of the SNX27 in the control of AQP2, further studies are needed to elucidate whether these two proteins bind to the same or overlapping sites and to define whether competition affects the trafficking and regulation of AQP2 abundance.

**Figure 11:**
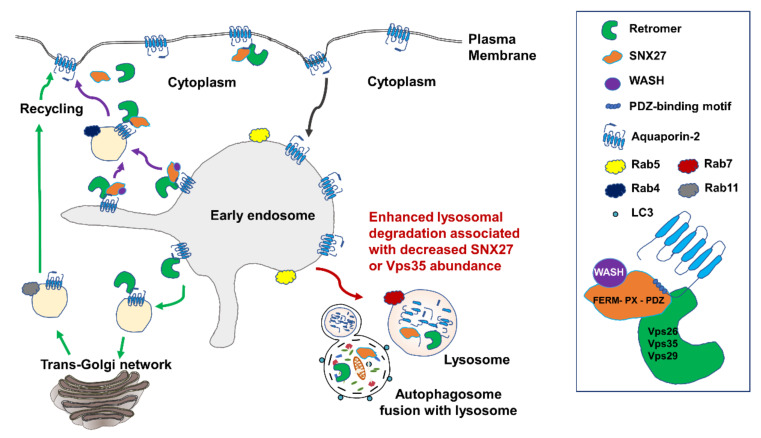
A summary of the findings in the present study. Upon the withdrawal of vasopressin stimulation, AQP2 is internalized into early endosomes for sorting. At the early endosome, AQP2 could be entered (1) to the recycling pathways either via the trans-Golgi network or directly to the plasma membrane, leading to AQP2 expression in the apical plasma membrane of the collecting duct principal cells in the kidney or (2) to the lysosomal pathway for degradation, leading to downregulation of AQP2 in the plasma membrane and the cells [1,2,5,6,7,8,9,12,61]. The class I PDZ domain-binding motif (X-[S/T]-X-Φ) in the carboxyl terminus of AQP2 is recognized by PDZ domain-containing proteins, e.g., SNX27. SNX27 is bound to the Vps26 of retromer complex subunits and concurrently binds to the PDZ ligand in its cargo proteins (AQP2); thereby, it could be involved in the recycling of AQP2 into the plasma membrane. The retromer complex is involved in the retrograde transport of proteins from endosomes to the trans-Golgi network. Alternatively, AQP2 could be sorted into lysosomes and subjected to lysosomal degradation to a greater extent upon the suppression of SNX27, i.e., SNX27 knockdown or deletion of the PDZ domain and Vps35 depletion [23].

Immunocytochemistry further demonstrated the co-localization of SNX27 and AQP2 in the HeLa cells co-transfected with FLAG-tagged SNX27 and HA-tagged AQP2. HeLa cells were used due to a relatively high transfection efficiency with the cationic lipids Lipofectamine 2000 and an easy and rapid growth [45]. Specifically, immunocytochemistry showed the diffuse co-localization of AQP2 with SNX27 throughout the cytoplasm. These findings suggested that SNX27 co-localizes with AQP2 in the kidney, and the PDZ domain of SNX27 importantly interacts with AQP2. In contrast, immunocytochemistry showed that AQP2 and SNX27 were localized in a similar manner (Figure 2M–O), despite the deletion of the PDZ domain in SNX27. However, when the PDZ domain was deleted in SNX27 (SNX27-ΔPDZ), AQP2 and SNX27 co-labeled differently: Expressed in a punctate pattern, and more eccentrically localized in the perinuclear region of the HeLa cells (Figure 2M–O). In Figure 3, we demonstrated that the observed punctate HA-tagged AQP2 labeling was intensively overlaid by Lysotracker staining when the PDZ domain was deleted in SNX27. Moreover, SNX27 knockdown was associated with an induction of autophagic flux and the formation of autolysosomes in mpkCCDc14 cells (Figure 9). Based on these results, we speculated that the observed punctate co-labeling of FLAG-tagged SNX27 lacking the PDZ domain and HA-tagged AQP2 (Figure 2M–O and Figure 3G,H) was associated with a lysosomal compartment in the cells (Figure 11) rather than the physiological interaction through the PDZ domain.

It has previously been demonstrated that SNX27 cooperates with the retromer complex and plays a role in the regulation of endosomal recycling and protein abundance [27,29]. In particular, Steinberg et al. [27] demonstrated that the PDZ domain of the SNX27 interacts directly with the retromer subunit Vps26 of the Vps26:Vps29:Vps35 trimer, which is necessary and sufficient to prevent lysosomal degradation of SNX27 cargo. We also demonstrated the complex formation between SNX27 and Vps35 by the immunoprecipitation assay in Figure 1E. Unpredictably, however, our data of the cell surface biotinylation assay and immunocytochemistry showed that dDAVP-induced AQP2 translocation to the apical plasma membrane was unaffected in mpkCCDc14 cells with siRNA-mediated SNX27 knockdown. Although siRNA transfection did not result in the complete abolition of SNX27 protein expression, this finding diverged from our previous study demonstrating that the depletion of Vps35, the cargo-selective trimer in the retromer complex, was associated with a decrease in dDAVP-responsive AQP2 trafficking to the apical plasma membrane [23]. The retromer complex contributes to the cargo sorting in the early endosomes and cargo delivery to the recycling endosome, resulting in the trafficking of proteins into the plasma membrane [21,22]. A recent study also demonstrated that AQP2 interacts with Vps35 and Rab7 [24], which is consistent with our previous findings [23]. SNX27, a retromer-associated cargo-binding protein, serves as a cargo selector for the retromer complex. SNX27 is bound to the Vps26 of retromer complex subunits and concurrently binds to the PDZ ligand in its cargo proteins, thereby it could be involved in the recycling of cargo proteins into the plasma membrane [26,27,30]. However, the cell surface biotinylation assay and immunocytochemistry in the present study suggested that retromer-dependent AQP2 trafficking is unlikely to require SNX27. This is supported by a previous study demonstrating that the retrograde cargo trafficking pathway mediated by the retromer complex requires SNX3 but not SNX27 or SNX-BAR proteins [46]. Another study also showed that mutation of the PDZ ligand in the parathyroid hormone receptor reduced its affinity for retromer, but it did not change the rate of recycling to the cell surface [29]. Thus, the observed intact dDAVP-induced AQP2 trafficking to the cell surface in the cells with SNX27 knockdown could suggest that other pathways are more importantly involved in AQP2 trafficking, e.g., protein kinase A-induced AQP2 phosphorylation, RhoA phosphorylation, intracellular calcium mobilization, and actin depolymerization [1,6,8]. 

However, it should be emphasized that the efficiency of the SNX27 silencing in our study was not sufficient (~30–50% of control) to draw a solid conclusion. Nevertheless, since AQP2 protein expression levels are very low in mpkCCDc14 cells under the basal condition, a period of time was required for inducing AQP2 protein expression for studying the effects of SNX27 knockdown on AQP2 expression. Therefore, due to the requirement of induction of cell polarity and AQP2 protein expression levels in mpkCCDc14 cells, the effects of siRNA treatment were studied 10 days after siRNA transfection in Figure 6, Figure 7, Figure 8. However, even though 10 days have passed after siRNA transfection, the protein abundance of SNX27 was still decreased by 30–50%, and importantly dDAVP-mediated AQP2 abundance was persistently and significantly downregulated compared to control siRNA-treated cells. We exploited the reverse transfection protocol, as we used in our previous studies [23,38], due to the low efficiency of epithelial cells in the conventional transfection protocol using cationic lipid-based transfectant. Besides, by comparison to the results obtained 10 days after siRNA transfection (~30–50% reduction of SNX27 protein abundance in Figure 6, Figure 7 and Figure 8), we also examined the changes of SNX27 protein abundance after the 4-day siRNA (50 nM) treatment in mpkCCDc14 cells (Figure 9). The results showed that treatment of the same amount of siRNA (50 nM) resulted in a further decrease in the protein abundance of SNX27 at day 4 (33 ± 1% of control, *p* < 0.05), i.e., a reduction of almost 70% protein abundance (Figure 9H,I). Thus, the effects of siRNA against SNX27 were getting weaker over 10 days after the reverse transfection. Further studies using a model of complete knockout of SNX27 are needed to fully understand the role of SNX27 in the retromer-dependent AQP2 regulation.

In contrast, semiquantitative immunoblotting revealed that dDAVP-induced AQP2 upregulation was significantly blunted after SNX27 knockdown in mpkCCDc14 cells. The changes in AQP2 protein abundance were not accompanied by the changes in AQP2 mRNA expression, suggesting that the SNX27-retromer complex is not likely to be involved in the regulation of *Aqp2* gene transcription. Thus, we speculated that SNX27 depletion could be associated with the diminished protein half-life due to enhanced degradation of AQP2 via lysosome and/or proteasome. Several studies have previously demonstrated that the depletion of retromer increases the lysosomal turnover of proteins [27,47,48,49], and we also previously demonstrated that depletion of the Vps35 subunit increases lysosomal degradation of AQP2 protein [23]. As described, SNX27 cooperates with the Vps26 subunit of the retromer complex, preventing the entry of transmembrane proteins into the lysosomal pathway and activating the retromer-tubule-based recycling to the plasma membrane [31]. In particular, SNX27 contains a PDZ domain that links membrane proteins having a class I PDZ domain-binding motif (X-[S/T]-X-Φ) at the carboxyl terminus to the retromer complex [20,50]. Since AQP2 has a PDZ ligand at the carboxyl terminus, AQP2 might be mis-sorted into lysosomes and subjected to lysosomal degradation to a greater extent upon the suppression of SNX27, i.e., SNX27 knockdown or deletion of the PDZ domain. Actually, we demonstrated that dDAVP-induced AQP2 upregulation was seen to a lesser extent in the cells with SNX27 knockdown. Moreover, the downregulation of AQP2 expression was observed to a greater extent during the withdrawal period of dDAVP stimulation under the SNX27 knockdown. Importantly, the downregulation of AQP2 was significantly inhibited by lysosomal inhibitor (chloroquine or bafilomycin) treatment but not by proteasomal inhibitor (MG132) treatment. Thus, AQP2 would be subjected to lysosomal degradation to a greater extent upon the suppression of SNX27, similar to the Vps35 depletion [23]. Consistently, Steinberg et al. [27] demonstrated the increased degradation of the glucose transporter GLUT1, the monocarboxylate transporter MCT1, and the Menkes disease copper transporter ATP7A in SNX27- or Vps35-depleted cells. Another example was that neuroligin 2 (NLG2) protein abundance was decreased in neurons with depletion of SNX27, which was mediated by enhanced lysosomal degradation [48]. Moreover, the alanine-, serine-, and cysteine-preferring transporter 2 (ASCT2, SLC1A5) was also demonstrated to be co-localized with lysosomal-associated membrane protein 1 (LAMP1), a lysosome marker in a punctate pattern in SNX27-knockout cells [51]. Specifically, we demonstrated that the observed punctate labeling of FLAG-tagged SNX27 and HA-tagged AQP2 was associated with a lysosomal compartment in the cells when the PDZ domain was deleted in SNX27.

The autophagy–lysosomal pathway is critically involved in protein degradation and clearance in eukaryotic cells. The degradation of cytoplasmic proteins is largely mediated by autophagy, involving the generation of autophagosomes, which fuse with lysosomes to form autolysosomes [52]. A previous study demonstrated that SNX27 knockout was associated with enhanced autophagy due to a decreased activation of the mammalian target of rapamycin (mTOR) complex 1 pathway [51]. Thus, we attempted to examine whether SNX27 knockdown induces the fusion of autophagosomes with lysosomes in mpkCCDc14 cells in vitro, by transfecting RFP-GFP-LC3 plasmid into the cells. The results showed that siRNA-mediated SNX27 knockdown induced the fusion of autophagosomes with lysosomes. Lithium has widely been used for the treatment of bipolar affective disorders; however, it is complicated by the development of nephrogenic diabetes insipidus (NDI) [53]. We and other researchers have demonstrated that lithium treatment induces a significant downregulation of AQP2 in the collecting duct principal cells, polyuria, and principal cell proliferation [40,54,55,56]. Since lithium is a potent autophagy inducer in many cell types [57], we examined the changes of SNX27 protein abundance in the kidneys of rats with lithium-induced NDI. Interestingly, SNX27 protein abundance was significantly decreased in the kidney inner medulla of rats with lithium-induced NDI, suggesting that autophagy could, at least in part, be involved in the downregulation of AQP2 in NDI, as demonstrated in the hypokalemia [58] or hypercalcemia model [59]. Previous studies have demonstrated that downregulation of AQP2 in lithium-induced NDI is mainly attributed to the inhibition of the vasopressin-induced adenylyl cyclase activity and cyclic adenosine monophosphate (cAMP) levels in the collecting duct [60]. However, the remodeling of the collecting duct, i.e., the observed reduction in principal cell numbers, in lithium-induced NDI could also contribute to the downregulation of AQP2 as well as SNX27 [56]. Further studies are therefore needed to clarify the role of SNX27, retromer complex, and autophagy in NDI conditions.

In summary, the PDZ domain-containing SNX27 interacts with AQP2 and regulates the stability of AQP2 protein through the regulation of the autophagy-lysosomal degradation of AQP2 (Figure 11). Acquired NDI induced by lithium treatment in rats is associated with a decreased SNX27 abundance in the kidneys, and further studies are warranted to examine the role of retromer–SNX27 and autophagy in NDI.

## Figures and Tables

**Table 1:** Primer sequences.

Construct		pGEX-4T-1 Vector Primer Sequence (5′-3′)	p3XFLAG-CMV-10 Vector Primer Sequence (5′-3′)
SNX-Full Length	F	GGTTCCGCGTGGATCCATGGCGGACGAGGACGGG	TGACGATGACAAGCTTATGGCGGACGAGGACGGG
	R	GATGCGGCCGCTCGAGCTAGGTGGCCACATCCCT	TCGCGGCCGCAAGCTTCTAGGTGGCCACATCCCT
SNX27-Δ(PX+FERM)	F	GGTTCCGCGTGGATCCATGGCGGACGAGGACGGG	TGACGATGACAAGCTTATGGCGGACGAGGACGGG
	R	GATGCGGCCGCTCGAGCTATGTGTAATCATAAAATGATTG	TCGCGGCCGCAAGCTTCTATGTGTAATCATAAAATGATTGTCCCAAG
SNX27-ΔFERM	F	GGTTCCGCGTGGATCCATGGCGGACGAGGACGGG	TGACGATGACAAGCTTATGGCGGACGAGGACGGG
	R	GATGCGGCCGCTCGAGCTAATTCTCATCAGATTCTGACAG	TCGCGGCCGCAAGCTTCTAATTCTCATCAGATTCTGACAGGAAC
SNX27-ΔPDZ	F	GGTTCCGCGTGGATCCAAGCAAGCAGTGCCCATA	TGACGATGACAAGCTTATGAAGCAAGCAGTGCCCATA
	R	GATGCGGCCGCTCGAGCTAGGTGGCCACATCCCT	TCGCGGCCGCAAGCTTCTAGGTGGCCACATCCCT

## References

[1] Jung H.J., Kwon T.H. (2016). Molecular mechanisms regulating aquaporin-2 in kidney collecting duct. Am. J. Physiol. Renal Physiol..

[2] Knepper M.A., Kwon T.H., Nielsen S. (2015). Molecular physiology of water balance. N. Engl. J. Med..

[3] Fenton R.A., Pedersen C.N., Moeller H.B. (2013). New insights into regulated aquaporin-2 function. Curr. Opin. Nephrol. Hypertens..

[4] Promeneur D., Kwon T.H., Frokiaer J., Knepper M.A., Nielsen S. (2000). Vasopressin V(2)-receptor-dependent regulation of AQP2 expression in Brattleboro rats. Am. J. Physiol. Renal Physiol..

[5] Ranieri M., Di Mise A., Tamma G., Valenti G. (2019). Vasopressin-aquaporin-2 pathway: Recent advances in understanding water balance disorders. F1000Research.

[6] Kwon T.H., Nielsen J., Moller H.B., Fenton R.A., Nielsen S., Frokiaer J. (2009). Aquaporins in the kidney. Handb. Exp. Pharmacol..

[7] Hoffert J.D., Fenton R.A., Moeller H.B., Simons B., Tchapyjnikov D., McDill B.W., Yu M.J., Pisitkun T., Chen F., Knepper M.A. (2008). Vasopressin-stimulated increase in phosphorylation at Ser269 potentiates plasma membrane retention of aquaporin-2. J. Biol. Chem..

[8] Nedvetsky P.I., Tamma G., Beulshausen S., Valenti G., Rosenthal W., Klussmann E. (2009). Regulation of aquaporin-2 trafficking. Handb. Exp. Pharmacol..

[9] Cheung P.W., Bouley R., Brown D. (2020). Targeting the Trafficking of Kidney Water Channels for Therapeutic Benefit. Annu. Rev. Pharmacol. Toxicol..

[10] Jung H.J., Kwon T.H. (2019). New insights into the transcriptional regulation of aquaporin-2 and the treatment of X-linked hereditary nephrogenic diabetes insipidus. Kidney Res. Clin. Pract..

[11] Sandoval P.C., Slentz D.H., Pisitkun T., Saeed F., Hoffert J.D., Knepper M.A. (2013). Proteome-wide measurement of protein half-lives and translation rates in vasopressin-sensitive collecting duct cells. J. Am. Soc. Nephrol..

[12] Moeller H.B., Aroankins T.S., Slengerik-Hansen J., Pisitkun T., Fenton R.A. (2014). Phosphorylation and ubiquitylation are opposing processes that regulate endocytosis of the water channel aquaporin-2. J. Cell Sci..

[13] Wu Q., Moeller H.B., Stevens D.A., Sanchez-Hodge R., Childers G., Kortenoeven M.L.A., Cheng L., Rosenbaek L.L., Rubel C., Patterson C. (2018). CHIP Regulates Aquaporin-2 Quality Control and Body Water Homeostasis. J. Am. Soc. Nephrol..

[14] Lee Y.J., Lee J.E., Choi H.J., Lim J.S., Jung H.J., Baek M.C., Frokiaer J., Nielsen S., Kwon T.H. (2011). E3 ubiquitin-protein ligases in rat kidney collecting duct: Response to vasopressin stimulation and withdrawal. Am. J. Physiol. Renal Physiol..

[15] Noda Y., Horikawa S., Furukawa T., Hirai K., Katayama Y., Asai T., Kuwahara M., Katagiri K., Kinashi T., Hattori M. (2004). Aquaporin-2 trafficking is regulated by PDZ-domain containing protein SPA-1. FEBS Lett..

[16] Boone M., Deen P.M. (2008). Physiology and pathophysiology of the vasopressin-regulated renal water reabsorption. Pflugers Arch..

[17] Moeller H.B., Olesen E.T., Fenton R.A. (2011). Regulation of the water channel aquaporin-2 by posttranslational modification. Am. J. Physiol. Renal Physiol..

[18] Kuwahara M., Asai T., Terada Y., Sasaki S. (2005). The C-terminal tail of aquaporin-2 determines apical trafficking. Kidney Int..

[19] Wang P.J., Lin S.T., Liu S.H., Kuo K.T., Hsu C.H., Knepper M.A., Yu M.J. (2017). Vasopressin-induced serine 269 phosphorylation reduces Sipa1l1 (signal-induced proliferation-associated 1 like 1)-mediated aquaporin-2 endocytosis. J. Biol. Chem..

[20] Seaman M.N. (2012). The retromer complex—Endosomal protein recycling and beyond. J. Cell Sci..

[21] Hierro A., Rojas A.L., Rojas R., Murthy N., Effantin G., Kajava A.V., Steven A.C., Bonifacino J.S., Hurley J.H. (2007). Functional architecture of the retromer cargo-recognition complex. Nature.

[22] Abubakar Y.S., Zheng W., Olsson S., Zhou J. (2017). Updated Insight into the Physiological and Pathological Roles of the Retromer Complex. Int. J. Mol. Sci..

[23] Lee M.S., Choi H.J., Park E.J., Park H.J., Kwon T.H. (2016). Depletion of vacuolar protein sorting-associated protein 35 is associated with increased lysosomal degradation of aquaporin-2. Am. J. Physiol. Renal Physiol..

[24] Wang W.L., Su S.H., Wong K.Y., Yang C.W., Liu C.F., Yu M.J. (2020). Rab7 involves Vps35 to mediate AQP2 sorting and apical trafficking in the collecting duct cells. Am. J. Physiol. Renal Physiol..

[25] Van Weering J.R., Cullen P.J. (2014). Membrane-associated cargo recycling by tubule-based endosomal sorting. Semin. Cell Dev. Biol..

[26] Ghai R., Bugarcic A., Liu H., Norwood S.J., Skeldal S., Coulson E.J., Li S.S., Teasdale R.D., Collins B.M. (2013). Structural basis for endosomal trafficking of diverse transmembrane cargos by PX-FERM proteins. Proc. Natl. Acad. Sci. USA.

[27] Steinberg F., Gallon M., Winfield M., Thomas E.C., Bell A.J., Heesom K.J., Tavare J.M., Cullen P.J. (2013). A global analysis of SNX27-retromer assembly and cargo specificity reveals a function in glucose and metal ion transport. Nat. Cell Biol..

[28] Temkin P., Lauffer B., Jager S., Cimermancic P., Krogan N.J., von Zastrow M. (2011). SNX27 mediates retromer tubule entry and endosome-to-plasma membrane trafficking of signalling receptors. Nat. Cell Biol..

[29] McGarvey J.C., Xiao K., Bowman S.L., Mamonova T., Zhang Q., Bisello A., Sneddon W.B., Ardura J.A., Jean-Alphonse F., Vilardaga J.P. (2016). Actin-Sorting Nexin 27 (SNX27)-Retromer Complex Mediates Rapid Parathyroid Hormone Receptor Recycling. J. Biol. Chem..

[30] Gallon M., Clairfeuille T., Steinberg F., Mas C., Ghai R., Sessions R.B., Teasdale R.D., Collins B.M., Cullen P.J. (2014). A unique PDZ domain and arrestin-like fold interaction reveals mechanistic details of endocytic recycling by SNX27-retromer. Proc. Natl. Acad. Sci. USA.

[31] Shinde S.R., Maddika S. (2017). PTEN Regulates Glucose Transporter Recycling by Impairing SNX27 Retromer Assembly. Cell Rep..

[32] Singh V., Yang J., Cha B., Chen T.E., Sarker R., Yin J., Avula L.R., Tse M., Donowitz M. (2015). Sorting nexin 27 regulates basal and stimulated brush border trafficking of NHE3. Mol. Biol. Cell.

[33] Choi H.J., Jung H.J., Kwon T.H. (2015). Extracellular pH affects phosphorylation and intracellular trafficking of AQP2 in inner medullary collecting duct cells. Am. J. Physiol. Renal Physiol..

[34] Chou C.L., Christensen B.M., Frische S., Vorum H., Desai R.A., Hoffert J.D., de Lanerolle P., Nielsen S., Knepper M.A. (2004). Non-muscle myosin II and myosin light chain kinase are downstream targets for vasopressin signaling in the renal collecting duct. J. Biol. Chem..

[35] Nielsen J., Kwon T.H., Frokiaer J., Knepper M.A., Nielsen S. (2007). Maintained ENaC trafficking in aldosterone-infused rats during mineralocorticoid and glucocorticoid receptor blockade. Am. J. Physiol. Renal Physiol..

[36] Song K., Gras C., Capin G., Gimber N., Lehmann M., Mohd S., Puchkov D., Rodiger M., Wilhelmi I., Daumke O. (2019). A SEPT1-based scaffold is required for Golgi integrity and function. J. Cell Sci..

[37] Lee S., Chang J., Blackstone C. (2016). FAM21 directs SNX27-retromer cargoes to the plasma membrane by preventing transport to the Golgi apparatus. Nat. Commun..

[38] Jung H.J., Kim S.Y., Choi H.J., Park E.J., Lim J.S., Frokiaer J., Nielsen S., Kwon T.H. (2015). Tankyrase-mediated beta-catenin activity regulates vasopressin-induced AQP2 expression in kidney collecting duct mpkCCDc14 cells. Am. J. Physiol. Renal Physiol..

[39] Kim J.E., Jung H.J., Lee Y.J., Kwon T.H. (2015). Vasopressin-regulated miRNAs and AQP2-targeting miRNAs in kidney collecting duct cells. Am. J. Physiol. Renal Physiol..

[40] Tingskov S.J., Hu S., Frokiaer J., Kwon T.H., Wang W., Norregaard R. (2018). Tamoxifen attenuates development of lithium-induced nephrogenic diabetes insipidus in rats. Am. J. Physiol. Renal Physiol..

[41] Tingskov S.J., Kwon T.H., Frokiaer J., Norregaard R. (2018). Tamoxifen Decreases Lithium-Induced Natriuresis in Rats With Nephrogenic Diabetes Insipidus. Front Physiol..

[42] Kimura S., Noda T., Yoshimori T. (2007). Dissection of the autophagosome maturation process by a novel reporter protein, tandem fluorescent-tagged LC3. Autophagy.

[43] Gong R., Wang P., Dworkin L. (2016). What we need to know about the effect of lithium on the kidney. Am. J. Physiol. Renal Physiol..

[44] Motoi Y., Shimada K., Ishiguro K., Hattori N. (2014). Lithium and autophagy. ACS Chem. Neurosci..

[45] Stephenson E.M. (1982). Locomotory invasion of human cervical epithelium and avian fibroblasts by HeLa cells in vitro. J. Cell Sci..

[46] Cui Y., Carosi J.M., Yang Z., Ariotti N., Kerr M.C., Parton R.G., Sargeant T.J., Teasdale R.D. (2019). Retromer has a selective function in cargo sorting via endosome transport carriers. J. Cell Biol..

[47] Wang W., Wang X., Fujioka H., Hoppel C., Whone A.L., Caldwell M.A., Cullen P.J., Liu J., Zhu X. (2016). Parkinson’s disease-associated mutant VPS35 causes mitochondrial dysfunction by recycling DLP1 complexes. Nat. Med..

[48] Binda C.S., Nakamura Y., Henley J.M., Wilkinson K.A. (2019). Sorting nexin 27 rescues neuroligin 2 from lysosomal degradation to control inhibitory synapse number. Biochem. J..

[49] Kvainickas A., Orgaz A.J., Nagele H., Diedrich B., Heesom K.J., Dengjel J., Cullen P.J., Steinberg F. (2017). Retromer- and WASH-dependent sorting of nutrient transporters requires a multivalent interaction network with ANKRD50. J. Cell Sci..

[50] Clairfeuille T., Mas C., Chan A.S., Yang Z., Tello-Lafoz M., Chandra M., Widagdo J., Kerr M.C., Paul B., Merida I. (2016). A molecular code for endosomal recycling of phosphorylated cargos by the SNX27-retromer complex. Nat. Struct. Mol. Biol..

[51] Yang Z., Follett J., Kerr M.C., Clairfeuille T., Chandra M., Collins B.M., Teasdale R.D. (2018). Sorting nexin 27 (SNX27) regulates the trafficking and activity of the glutamine transporter ASCT2. J. Biol. Chem..

[52] Klionsky D.J., Emr S.D. (2000). Autophagy as a regulated pathway of cellular degradation. Science.

[53] Timmer R.T., Sands J.M. (1999). Lithium intoxication. J. Am. Soc. Nephrol..

[54] Kwon T.H., Laursen U.H., Marples D., Maunsbach A.B., Knepper M.A., Frokiaer J., Nielsen S. (2000). Altered expression of renal AQPs and Na(+) transporters in rats with lithium-induced NDI. Am. J. Physiol. Renal Physiol..

[55] Nielsen J., Kwon T.H., Christensen B.M., Frokiaer J., Nielsen S. (2008). Dysregulation of renal aquaporins and epithelial sodium channel in lithium-induced nephrogenic diabetes insipidus. Semin. Nephrol..

[56] Christensen B.M., Kim Y.H., Kwon T.H., Nielsen S. (2006). Lithium treatment induces a marked proliferation of primarily principal cells in rat kidney inner medullary collecting duct. Am. J. Physiol. Renal Physiol..

[57] Bao H., Zhang Q., Liu X., Song Y., Li X., Wang Z., Li C., Peng A., Gong R. (2019). Lithium targeting of AMPK protects against cisplatin-induced acute kidney injury by enhancing autophagy in renal proximal tubular epithelial cells. FASEB J..

[58] Khositseth S., Uawithya P., Somparn P., Charngkaew K., Thippamom N., Hoffert J.D., Saeed F., Michael Payne D., Chen S.H., Fenton R.A. (2015). Autophagic degradation of aquaporin-2 is an early event in hypokalemia-induced nephrogenic diabetes insipidus. Sci. Rep..

[59] Khositseth S., Charngkaew K., Boonkrai C., Somparn P., Uawithya P., Chomanee N., Payne D.M., Fenton R.A., Pisitkun T. (2017). Hypercalcemia induces targeted autophagic degradation of aquaporin-2 at the onset of nephrogenic diabetes insipidus. Kidney Int..

[60] Christensen S., Kusano E., Yusufi A.N., Murayama N., Dousa T.P. (1985). Pathogenesis of nephrogenic diabetes insipidus due to chronic administration of lithium in rats. J. Clin. Investig..

[61] Nedvetsky P.I., Stefan E., Frische S., Santamaria K., Wiesner B., Valenti G., Hammer J.A., Nielsen S., Goldenring J.R., Rosenthal W. (2007). A Role of myosin Vb and Rab11-FIP2 in the aquaporin-2 shuttle. Traffic.

